# Supplementary material for: Factors Associated with Post-Transplant Active Epstein-Barr Virus Infection and Lymphoproliferative Disease in Hematopoietic Stem Cell Transplant Recipients: A Systematic Review and Meta-Analysis
Source: Vaccines (Basel). 2021 Mar 19;9(3):288. doi: 10.3390/vaccines9030288 (PMC8003684; doi:10.3390/vaccines9030288)
Supplement: Supplementary file 1 [file vaccines-09-00288-s001.pdf]

Supplementary materials: Table S1, Table S2a, Table S2b, TableS3 and Table S4

Table S1: Search equation used in Medline and EMBASE

Medline: search was performed using the following equation: **(EBV*.mp. or HHV4*.mp. or Epstein-Barr virus*.mp. or exp Herpesvirus 4, Human/ or exp Epstein-Barr Virus Infections/) AND (risk*.mp. or reactivation*.mp or active*.mp. or chronic active*.mp. or activation*.mp. or viremia*.mp. or DNAemia*.mp. or infection*.mp. or posttranspl*.mp. or post-transpl*.mp. or lymphopro*.mp. or PTLD.mp. or exp Risk/ or exp Risk Factors/ or exp Virus Activation/ or exp Viremia/ or exp Infection/ or exp Lymphoproliferative Disorders/) AND (exp Stem Cell Transplantation/ or exp Cord Blood Stem Cell Transplantation/ or exp Hematopoietic Stem Cell Transplantation/ or exp Hematopoietic Stem Cells/ or exp Peripheral Blood Stem Cell Transplantation/ or exp Bone Marrow Transplantation/).**

EMBASE: search was performed using the following equation: (EBV*.mp. or HHV4*.mp. or Epstein-Barr virus*.mp. or exp Epstein-Barr Virus / or exp Epstein-Barr Virus Infections/) AND (risk*.mp. or reactivation*.mp or active*.mp. or chronic active*.mp. or activation*.mp. or viremia*.mp. or DNAemia*.mp. or infection*.mp. or posttranspl*.mp. or post-transpl*.mp. or lymphopro*.mp. or PTLD.mp. or exp Risk/ or exp Risk Factors/ or exp recurrence risk/ or exp Virus reactivation/ or exp Viremia/ or exp Infection/ or exp posttransplant lymphoproliferative disease/) AND (exp hematopoietic stem cell transplantation/ or exp Stem Cell Transplantation/ or exp allogeneic hematopoietic stem cell transplantation or exp bone marrow transplantation/ or exp allogenic bone marrow transplantation/ or exp peripheral blood stem cell transplantation/ or exp allogeneic peripheral blood stem cell transplantation/ or exp cord blood stem cell transplantation)**.**

**Limits**	**Medline**	**EMBASE**
**Language**	English or French	
**Year of publication**	1946-June 2020	1974-June 2020
**Type of publication**	Journal article	Article

Table S2a: COMPONENT RATINGS OF STUDY (*a modified version of the Effective Public Health Practice Project (EPHPP) Quality Assessment Tool for Quantitative Studies*[24,25])

SELECTION BIAS

Strong: was assigned to a prospective cohort study or randomised control trial (RCT), if the retention of subjects in the study was not likely to be dependent on both exposure and outcome. Retention was evaluated within a follow-up of at least 3-6 months (*). In the case of a retrospective cohort study, this qualifier was assigned if the enrollment of subjects in the study was not likely to be related to both exposure and outcome and if the retention of subjects was not likely to be dependent on both exposure and outcome.
For the other used design (case-control study only), this qualifier was assigned if the cases and controls included were representative of the population and if the participation rate was not differential.
Moderate: was assigned to a prospective cohort study or RCT, if the retention of subjects within 3-6 months of follow-up in the study was somewhat likely to be dependent on both exposure and outcome. In the case of a retrospective cohort study, this qualifier was assigned if the enrollment of subjects in the study was somewhat likely to be related to both exposure and outcome or if the retention of subjects was somewhat likely to be dependent on both exposure and outcome. For the other used design (case-control study only), this qualifier was assigned if the cases and controls included were representative of the population and if the participation rate was differential.
Weak: was assigned to a prospective cohort study or RCT, if the retention of subjects within 3-6 months of follow-up in the study was very likely to be dependent on both exposure and outcome. In the case of a retrospective cohort study, this qualifier was assigned if the enrollment of subjects in the study was very likely to be related to both exposure and outcome or if the enrollment of subjects in the study was not described or if the retention of subjects was very likely to be dependent on both exposure and outcome or if the retention was not described. For the other design found (case-control study only), this qualifier was assigned if the cases and controls included were not representative of the population and if the participation rate was differential.

(*) During the first 3 months post-transplant most patients are usually still followed.

STUDY DESIGN

Strong: was assigned to a cohort study, randomised control trial (RCT)
Moderate: was assigned to a case-control study.
Weak: was assigned to a study that did not state the design used.

CONFOUNDERS

Strong: was assigned to a study that controlled for confounding bias. Specifically, a method to control for confounders was applied (ex: confounders included in the multivariate analysis model or the distributions of the confounding factors were balanced between each group).
Moderate: was assigned to a study that did not specifically consider confounding bias, but where a multivariate analysis has been performed.
Weak: was assigned when there was no control for confounding.

DATA COLLECTION METHODS

Strong: was assigned if EBV infection testing was based on the PCR technique and if the same procedures (ie. blood compartment, laboratory techniques, threshold) were used for all patients. For studies with PTLD as outcome, this rating was given if the diagnosis of PTLD was made similarly for all patients and only proven cases of PTLD were considered.
Moderate: was assigned if EBV infection testing was based on the PCR technique but the procedures used were not the same for all patients. For studies with PTLD as outcome, this rating was given if the diagnosis of PTLD was made similarly for all patients and probable and proven cases of PTLD were considered or the methods used to diagnose PTLD were not the same for all patients.
Weak: was assigned if EBV infection testing was not based on the PCR technique or no information about the test used was available. For studies with PTLD as outcome, this rating was given if probable or proven PTLD and post-transplant EBV infection were combined to define the event of interest or no information about the method used to diagnose PTLD was available.

Overall rating for a study:
Strong: no Weak rating
Moderate: one Weak rating except for the component confounders
Weak: Weak ranting for the component confounders OR two or more Weak ratings for other components

Table S2b: Results of the quality evaluation of the 77 articles included in this systematic review

First author, year	Outcome	Component ratings				Overall rating
		Selection bias	Study design	Confounders	Data collection	
Ali, 2019[30]	**PTLD**	Weak	Strong	Weak	Moderate	Weak
Althubaiti, 2019[31]	**PTLD**	Weak	Strong	Weak	Moderate	Weak
Atay, 2018[32]	**EBV**	Strong	Strong	Weak	Strong	Weak
Auger, 2014 [33]	**EBV**	Strong	Strong	Weak	Strong	Weak
Bogunia-Kubik, 2007[34]	**EBV**	Strong	Strong	Strong	Strong	Strong
Bogunia-Kubik, 2005[35]	**EBV**	Strong	Strong	Strong	Strong	Strong
Bordon, 2012[36]	**EBV**	Weak	Strong	Moderate	Strong	Moderate
Brunstein, 2006[37]	**EBV/PTLD**	Strong	Strong	Strong	Weak	Moderate
Burns,2016[38]	**EBV**	Strong	Strong	Moderate	Strong	Strong
Buyck, 2009[39]	**PTLD**	Weak	Strong	Moderate	Strong	Moderate
Carpenter, 2010[22]	**EBV**	Strong	Strong	Moderate	Strong	Strong
Cesaro, 2004[40]	**EBV**	Weak	Strong	Moderate	Strong	Moderate
Cesaro, 2010[41]	**EBV**	Weak	Strong	Weak	Strong	Weak
Chiereghin, 2016[42]	**EBV**	Strong	Strong	Weak	Strong	Weak
Chiereghin, 2019[43]	**EBV**	Moderate	Strong	Weak	Strong	Weak
Christopeit, 2013[44]	**EBV**	Weak	Strong	Strong	Strong	Moderate
Cohen, 2005[45]	**EBV**	Weak	Strong	Moderate	Strong	Moderate
Cohen, 2005[45]	**PTLD**	Weak	Strong	Moderate	Strong	Moderate
Comoli,2007[46]	**EBV**	Strong	Strong	Weak	Strong	Weak
Czyżewski, 2019[47]	**EBV**	Moderate	Strong	Weak	Weak	Weak
D'Aveni, 2011[48]	**EBV**	Weak	Strong	Weak	Strong	Weak
Dumas, 2013[49]	**EBV**	Weak	Strong	Moderate	Moderate	Moderate
Düver, 2020[50]	**EBV**	Strong	Strong	Moderate	Moderate	Strong
Elmahdi, 2016[51]	**EBV**	Weak	Strong	Moderate	Strong	Moderate
Fan, 2016[52]	**EBV**	Weak	Strong	Moderate	Strong	Moderate
Figgins, 2019[53]	**EBV**	Strong	Strong	Weak	Strong	Weak
Fujimoto, 2019[54]	**PTLD**	Moderate	Strong	Moderate	Moderate	Strong
Gao, 2019[55]	**EBV**	Moderate	Strong	Moderate	Strong	Strong
Gao, 2019[55]	**PTLD**	Moderate	Strong	Moderate	Moderate	Strong
Garcia-Cadenas, 2015[56]	**EBV**	Moderate	Strong	Moderate	Strong	Strong
Garcia-Cadenas, 2015[56]	**PTLD**	Moderate	Strong	Moderate	Moderate	Strong
Han, 2014[57]	**EBV**	Moderate	Strong	Weak	Strong	Weak
Hiwarkar, 2013[58]	**EBV**	Weak	Strong	Moderate	Strong	Moderate
Hoegh-Petersen, 2011[59]	**PTLD**	Strong	Strong	Weak	Moderate	Weak
Hoshino, 2001[60]	**EBV**	Weak	Strong	Weak	Strong	Weak

Table S2b: Results of the quality evaluation of the 77 articles included in this systematic review

First author, year	Outcome	Component ratings				Overall rating
		Selection bias	Study design	Confounders	Data collection	
Islam,2010[61]	**EBV**	Moderate	Strong	Weak	Strong	Weak
Issa, 2019[62]	**EBV**	Moderate	Strong	Weak	Strong	Weak
Kutnik, 2019[63]	**EBV**	Strong	Strong	Weak	Weak	Weak
Jaskula, 2010[64]	**EBV**	Weak	Strong	Moderate	Strong	Moderate
Juvonen, 2007[65]	**EBV**	Moderate	Strong	Moderate	Strong	Strong
Kalra, 2018[66]	**PTLD**	Strong	Strong	Moderate	Moderate	Strong
Kullberg-Lindh, 2011[67]	**EBV**	Moderate	Strong	Moderate	Strong	Strong
Laberko,2017[68]	**EBV**	Strong	Strong	Moderate	Moderate	Strong
Landgren, 2009[69]	**PTLD**	Moderate	Strong	Moderate	Moderate	Strong
Li, 2018[70]	**EBV**	Strong	Strong	Weak	Weak	Weak
Lin, 2019[71]	**EBV**	Strong	Strong	Moderate	Strong	Strong
Liu, 2020[72]	**EBV**	Strong	Strong	Moderate	Strong	Strong
Liu, 2020[72]	**PTLD**	Moderate	Strong	Weak	Moderate	Weak
Liu, 2013[73]	**EBV**	Strong	Strong	Moderate	Strong	Strong
Liu, 2013[26]	**EBV**	Strong	Strong	Moderate	Strong	Strong
Liu, 2013[26]	**PTLD**	Strong	Strong	Moderate	Strong	Strong
Liu, 2018[74]	**EBV**	Moderate	Strong	Moderate	Strong	Strong
Marinho-Dias, 2019[75]	**EBV**	Strong	Strong	Moderate	Strong	Strong
Meijer, 2004[76]	**EBV**	Strong	Strong	Weak	Strong	Weak
Mountjoy, 2020[77]	**EBV**	Moderate	Strong	Weak	Strong	Weak
Neumann, 2018[78]	**EBV**	Moderate	Moderate	Moderate	Moderate	Strong
Nowak, 2019[79]	**EBV**	Moderate	Strong	Weak	Weak	Weak
Omar, 2009[80]	**EBV**	Weak	Strong	Moderate	Strong	Moderate
Pagliuca, 2019[81]	**PTLD**	Strong	Strong	Moderate	Moderate	Strong
Park, 2020[82]	**EBV**	Strong	Strong	Weak	Weak	Weak
Patriarca, 2013[4]	**EBV**	Strong	Strong	Moderate	Strong	Strong
Peric, 2012[83]	**EBV**	Strong	Strong	Weak	Strong	Weak
Peric, 2011[84]	**EBV**	Strong	Strong	Moderate	Strong	Strong
Ru, 2020[85]	**EBV**	Moderate	Strong	Moderate	Strong	Strong
Rustia, 2016[86]	**EBV**	Moderate	Strong	Weak	Strong	Weak
Sanz,2014[87]	**EBV**	Strong	Strong	Moderate	Strong	Strong
Sanz,2014[87]	**PTLD**	Strong	Strong	Moderate	Strong	Strong
Sirvent-von Bueltzingsloewen, 2002[88]	**EBV**	Strong	Strong	Moderate	Strong	Strong
Styczynski, 2013[89]	**PTLD**	Weak	Strong	Weak	Moderate	Weak
Torre-Cisneros, 2004[90]	**EBV**	Weak	Strong	Moderate	Strong	Moderate

Table S2b: Results of the quality evaluation of the 77 articles included in this systematic review

First author, year	Outcome	Component ratings				Overall rating
		Selection bias	Study design	Confounders	Data collection	
Trottier, 2012[91]	**EBV**	Weak	Strong	Strong	Moderate	Moderate
Tsoumakas, 2019[92]	**EBV**	Strong	Strong	Moderate	Strong	Strong
Uhlin, 2014[93]	**PTLD**	Moderate	Strong	Moderate	Strong	Strong
van der Velden, 2013[94]	**EBV/PTLD**	Strong	Strong	Moderate	Weak	Moderate
Van Esser, 2001[95]	**EBV**	Moderate	Strong	Moderate	Strong	Strong
Van Esser, 2001[95]	**PTLD**	Moderate	Strong	Moderate	Strong	Strong
Wang, 2019[96]	**EBV**	Moderate	Strong	Moderate	Strong	Strong
Xu, 2015[97]	**PTLD**	Moderate	Moderate	Moderate	Moderate	Strong
Xuan, 2012[98]	**EBV**	Strong	Strong	Strong	Strong	Strong
Xuan, 2013[16]	**PTLD**	Strong	Strong	Moderate	Moderate	Strong
Yu, 2019[99]	**EBV**	Moderate	Strong	Moderate	Weak	Moderate
Zallio, 2013[23]	**EBV**	Weak	Strong	Moderate	Strong	Moderate
Zhou, 2020[100]	**EBV**	Strong	Strong	Moderate	Strong	Strong
Zhou, 2020[101]	**PTLD**	Strong	Strong	Weak	Moderate	Weak
**Summary of the component rating**						
**Strong, n (%)**	**EBV**	**29 (46)**	**62 (98.4)**	**6 (9.5)**	**51 (81)**	**27 (42.9)**
**Moderate, n (%)**		**18 (28.6)**	**1 (1.6)**	**36 (57.1)**	**5 (7.9)**	**15 (23.8)**
**Weak, n (%)**		**16 (25.4)**	**0 (0)**	**21 (33.3)**	**7 (11.1)**	**21 (33.3)**
**Strong, n (%)**	**PTLD**	**8 (38.1)**	**20 (95.2)**	**0 (0)**	**6 (28.6)**	**12 (57.1)**
**Moderate, n (%)**		**8 (38.1)**	**1 (4.8)**	**15 (71.4)**	**14 (66.7)**	**3 (14.3)**
**Weak, n (%)**		**5 (23.8)**	**0 (0)**	**6 (28.6)**	**1 (4.8)**	**6 (28.6)**

Table S3: Characteristics of the 77 studies included in the systematic review

First author, year	Country (Date of graft)	Study type	Post-graft follow-up duration	Population				EBV DNAemia/PTLD definition	Frequency of testing	Blood compartment used for the test	Statistical methods
				Graft type	Sample	Age	Pediatrics /Adults				
Ali, 2019[30]	Canada (2006-2015)	Retrospective	NR	BM, PBSC, CB	408	No PTLD group Median 7.6 years (range: 0.1-17.8 years) PTLD group Median 5.9 years (range: 2.3-17.3 years)	Pediatrics	Proven PTLD	NA	NA	Fisher's exact test
Althubaiti, 2019[31]	Canada (January 2010-December 2016)	Retrospective	NR	BM, PB, CB	26	No PTLD group Median 7 years (range: 2-14 years) PTLD group Median 9 years (range: 2-17 years)	Pediatrics	Probable or proven PTLD	NA	NA	Chi2, Fisher's exact test and Mann-Whitney U test
Atay, 2018[32]	Turkey (January 2014 to September 2016)	Retrospective	Median: 14 months Range (1-31) months	BM, PBSC, CB	171	Median: 7.38 years Range: (0.4-18) years	Pediatrics	Not mentioned	Weekly during the post- transplant period on inpatients and outpatients when symptomatic.	Not mentioned	Chi2 test
Auger, 2014 [33]	France (NR)	Retrospective	36.6 months (95% IC 31.5–45.7).	PBSC, BM and UCB	190	Median 51 years Range: (18-69) years IQR: (38-58) years	Adults	EBV viral load superior or equal to 500 copies/mL, and increasing 1 week later	Weekly during the first 6 months and monthly thereafter.	Peripheral blood	Chi2 test, Wilcoxon nonparametric test, Kruskal-Wallis test
Bogunia-Kubik, 2007[34]	Poland (NR)	Retrospective	2-3 months	PBSC, BM	92	Median: 28.5 years Range: (0.3-60) years	Pediatrics & Adults	EBV viral load >10 EBV-DNA copies/10^5 cells	On average 2 measurements per patient performed 2-3 months post-transplant.	Peripheral blood	Fisher exact test for univariate analysis. Logistic regression for multivariate analysis
Bogunia-Kubik, 2005[35]	Poland (1997-2003)	Retrospective	2-3 months	PBSC, BM	83	Median: 25 years Range: (0.3-55) years	Pediatrics & Adults	EBV viral load >10 EBV-DNA copies/10^5 cells	On average 2 measurements per patient performed 2-3 months post-transplant.	Peripheral blood	Fisher exact test for univariate analysis. Logistic regression for multivariate analysis
Bordon, 2012[36]	Belgium (Jan 2002-Dec 2009)	Retrospective	1 year	PBSC, BM and UCB	80	Mean: 6.3 years Range: (0.2-19.4) years	Pediatrics	EBV viral load >300 copies/µg DNA	Biweekly during the first 3 months, then monthly until 1 year. In the case of a positive PCR test or if clinically indicated, test was repeated weekly.	Whole blood	Fisher exact test and Mann-Whitney U-test for univariate analysis. Logistic regression for multivariate analysis
Brunstein, 2006[37]	USA (July 1994-March 2005)	Multicenter retrospective	Median (range): 1.2 years (77 days-9.2 years)	UCB	335	Median: 16 years Range: (0.2-69) years	Pediatrics & Adults	EBV viremia was defined as more than 1000 copies of EBV DNA per ml of whole blood and EBV PTLD was defined as biopsy- or autopsy-proven post-transplantation lymphoma, or	NA	NA	Multivariate Cox regression

Table S3: Characteristics of the 77 studies included in the systematic review

First author, year	Country (Date of graft)	Study type	Post-graft follow-up duration	Population				EBV DNAemia/PTLD definition	Frequency of testing	Blood compartment used for the test	Statistical methods
				Graft type	Sample	Age	Pediatrics /Adults				
								viremia along with computerized tomography nodal or soft- tissue abnormalities consistent with PTLD.			
Burns, 2016[38]	United Kingdom, (May 2009 to September 2012)	Retrospective	Median 28 months	PBSC	186	Median 51 years Range: (17-71) years	Pediatrics & Adults	EBV-VL superior or equal to 500 genomes/m	Every 1-2 week(s) for the first 6 months and intermittently thereafter.	Whole blood	Univariate and multivariate analysis using Cox proportional hazards models
Buyck, 2009[39]	United Kingdom, (1989-2006)	Retrospective	NR	Allo-SCT	87	Median 20 years Range: (4-53) years	Pediatrics & Adults	EBV-PTLD was confirmed by radiological and/or histopathological evidence of lymphoproliferation with EBV confirmed either by PCR or immunohistochemistry.	NA	NA	Univariate and multivariate analysis using Cox proportional hazards models
Carpenter, 2010[22]	United Kingdom (May 2005-Sept 2009)	Retrospective	Median: 2.4 years	PBSC	111[a]	Median: 43 years Range: (16-67) years	Pediatrics & Adults	EBV viral load > 200 copies/mL	Weekly until the 100th day and then at follow-up by real-time quantitative polymerase chain reaction amplification of *EBNA 1* gene	NR	Fine Gray competitive risk model
Cesaro, 2004[40]	Italy (Jan 1998-Dec 2003)	Retrospective	180 days	BM, UCB	79[b]	Median: 9.6 years Range: (1.4-18) years	Pediatrics	Must involve at least two consecutive positive PCR results (EBV viral load ≥300 genome copies x 10^5 PBMC).	Weekly between the 15th and 100th days post-graft. Biweekly between the 101st and 180th days if clinically indicated	Peripheral blood	Chi2 test or Fisher's exact test and multivariate Cox models
Cesaro, 2010[41]	Italy (Jan 1998-Dec 2007)	Retrospective	180 days	BM, UCB	89	Median: 9 years Range: (0.7-18) years	Pediatrics	Defined at the first of at least two consecutive positive PCR results (EBV viral load ≥300 genome copies x 10^5 PBMC)	Weekly between the 15th and 100th days post-graft. Biweekly between the 101st and 180th days when clinically indicated	Peripheral blood	Chi2 test or Fisher's exact test
Chiereghin, 2016[42]	Italy (March 2012-Nov 2013)	Prospective	Median: 7.1 months Range:(1-22) months	PBSC, BM and UCB	28	Mean: 9.4 years Range: (9 months - 18.4 years)	Pediatrics	EBV viral load ≥10000 copies/mL	Weekly for the first 100 days post-transplant and biweekly until the 180th day. Subsequently the tests were performed when clinically indicated	Whole blood	Chi2 test
Chiereghin, 2019[43]	Italy (February 2014-February 2015)	Prospective	>2 months	BM, PB, CB	51	Adults Mean 40 years (range: 18-59 years) Pediatrics Mean 9 years (range: 9 months-17 years)	Pediatrics & Adults	EBV-DNA>500 copies/mL	Weekly for the first 100 days post-transplant and biweekly until the 180th day.	Whole blood	Chi2, Fisher's exact tests

Table S3: Characteristics of the 77 studies included in the systematic review

First author, year	Country (Date of graft)	Study type	Post-graft follow-up duration	Population				EBV DNAemia/PTLD definition	Frequency of testing	Blood compartment used for the test	Statistical methods
				Graft type	Sample	Age	Pediatrics /Adults				
Christopeit, 2013[44]	Germany (July 2005-Sept 2008)	Retrospective	>30 days	Allo-HSCT	28[c]	Median: 59.5 years Range: (22-70) years	Adults	EBV viral load ≥100 copies/mL	Biweekly during hospitalization and at each contact after discharge from the hospital	Peripheral blood	Multivariate logistic regression
Cohen, 2005[45]	United Kingdom (Jan 1999-Jun 2002)	Prospective	NR	BM, PBSC, BM+ PBSC, UCB	128	Median: 4.1 years Range: (0.2-17.7) years	Pediatrics	EBV-DNA detected in whole blood (positive) and next day plasma (semi-quantitative – approximately 100 DNA copies/mL in plasma – 10 EBV-DNA copies/cell)	Weekly until CD4$^+$ becomes > 0.3x10^9/ L	Whole blood for DNA detection and plasma for semi-quantitative if DNA positive	Univariate, bivariate and multivariate logistic regression. Each factor with a p-value <0.1 in univariate analysis was introduced in a bivariate model with the conditioning regime variable (RIC vs. CIC). Factors with a p-value <0.5 are considered in the multivariate model.
								EBV-LPD was subclassified clinically as either localized or disseminated and lymphadenopathic or lymphomatous, according to the Pittsburgh classification	NA	NA	
Comoli, 2007[46]	Italy, (August 2001 to February 2005)	Prospective	Median: 23 months	PBSC	27	Median: 8 years Range: (1-21) years	Pediatrics & Adults	EBV-DNA load above 1000 copies/10^5 PBMC or 1000 copies/10 uL whole blood associated in two consecutive samples	Weekly during first 3 months, and monthly thereafter until 1 year after transplantation	PBM (or whole blood if prior to hematopoietic reconstitution)	Univariate analysis using Chi-square test
Czyżewski, 2019[47]	Poland (January 2012-December 2015)	Multicenter retrospective study	NR	BM, PB, CB	1,569	NR	Pediatrics & Adults	EBV but not specified	Weekly	NR	Chi 2 test
D'Aveni, 2011[48]	France (January 2006-December 2006)	Retrospective	1-year	PBSC, BM and UCB	40[d]	Median 30 years Range: (0-64) years	Pediatrics & Adults	EBV DNA ≥1000 copies/mL	Twice a week during the first 3 months after transplantation and in case of DNAemia	Whole blood	Chi2 test
Dumas, 2013[49]	France (Jan 2003-Dec 2009)	Multicenter Retrospective	100 days[e]	UCBT	175	Median: 23 years Range: (0.6-64) years	Pediatrics & Adults	EBV viremia was defined as detection and quantification of EBV DNA in peripheral blood according to each transplant center RQ–PCR threshold	At least one test per week during the first 100 days post-transplant and thereafter when clinically indicated	Peripheral blood	The variables with a p-value <0.1 in univariate analysis were considered in the multivariate model of Fine Gray
Düver, 2020[50]	Germany (January 2005-December 2015)	Retrospective	Median 365 days (range: 22-365 days)	BM, PB, only one patient received CB	107	Median 9 years (range: 2 months-22.2 years)	Pediatrics	EBV-DNA>200 copies	One or twice a week in the first 40 days, weekly from day 40 to 60 and from day 60 on every second week until day 100.	Serum or plasma	Chi2, Fisher's exact test and binary logistic model. Only variables with p<0.20 were considered in binary logistic model.
Elmahdi, 2016[51]	Japan (July 1999-Nov 2011)	Retrospective	NR	BM, BM +PBSC, UCB	37	Median: 8 years Range: (1-19) years	Pediatrics	Peripheral virtual load >1 x 10$^{2.5}$ copies/µg DNA of peripheral blood	Weekly testing	Peripheral blood or whole blood	Univariate and multivariate Cox models. Only factors with a p-

Table S3: Characteristics of the 77 studies included in the systematic review

First author, year	Country (Date of graft)	Study type	Post-graft follow-up duration	Population				EBV DNAemia/PTLD definition	Frequency of testing	Blood compartment used for the test	Statistical methods
				Graft type	Sample	Age	Pediatrics /Adults				
								mononuclear cells or a viral load >20000 copies/mL in whole blood without the presence of symptoms			value <0.1 in univariate analysis were included in the multivariate model
Fan, 2016[52]	China (Jan 2012-June 2012)	Retrospective	12 months	PBSC, PBSC+ BM	44[f]	Median: 26 Range: (16-55) years	Pediatrics & Adults	NR	The tests were performed at least once a week during the first month, twice a week during the 2nd and 3rd months and then once or twice a month until December 2012	Plasma	Binary logistic regression
Figgins, 2019[53]	USA (March 2016-June 2017)	Retrospective	Median 12.8 months (range: 1.0-23.1 months)	HSCT	123	Range: 19-77 years	Adults	Positive EBV PCR test	NR	Serum	Log-rank test
Fujimoto, 2019[54]	Japan (January 1990-December 2016)	Multicenter retrospective	NR	BM, PB, CB	64,539	Range: 16-88 years	Pediatrics & Adults	Diagnosis of PTLD established by treating physicians and hematologic pathologists	NA	NA	Univariate and multivariate Cox model
Gao, 2019[55]	China (March 2014-December 2017)	Retrospective	The endpoint of follow-up was set for April 30, 2018 for all surviving subjects.	PBSC	200	Median 37 years (range: 7-63 years)	Pediatrics & Adults	EBV-VL≥1000 copies/mL	Weekly and the frequency of monitoring should be increased to twice a week in patients with rising DNA copies.	Plasma	Multivariate Fine and Gray model. Only variables with p<0.1 in univariate analysis were included in multivariate model.
								Proven or probable PTLD	NA	NA	
Garcia-Cadenas, 2015[56]	Spain (Sept 2006-May 2013)	Prospective	Follow-up was stopped after the first 6 months if no EBV reactivation occurred	UCB, PBSC, BM	93	Median: 41 years Range: (18-67) years	Adults	EBV PCR viral load in plasma above 1000 copies of DNA per mL	Performed weekly	Plasma	Variables with a p-value <0.1 in univariate analysis were considered in a multivariate Cox model. p-value<0.05 were considered statistically significant.
								Proven EBV-PTLD was defined as the histologically diagnosed PTLD with symptoms and/or signs from affected organ(s). Probable disease was defined as a typical clinical manifestation(s) of PTLD plus an EBV viral load >1000 copies per ml, in the absence of other causative factors or established diseases.	NA	NA	
Han, 2014[57]	Korea (January 2008-	Retrospective	6 months	BM, PBSC, CB	248	≤10 years group Median: 5 years	Pediatrics	EBV DNA >500 copies/mL at any time during the	The tests were initially performed 2 or 3 weeks after graft and then	Whole blood	Chi2 test

Table S3: Characteristics of the 77 studies included in the systematic review

First author, year	Country (Date of graft)	Study type	Post-graft follow-up duration	Population				EBV DNAemia/PTLD definition	Frequency of testing	Blood compartment used for the test	Statistical methods
				Graft type	Sample	Age	Pediatrics /Adults				
	March 2013)					Range: (0-10) years<br><br>11-20 years group<br>Median: 14 years<br>Range: (11-20) years		first 6 months after graft	routinely at 1, 3, and 6 months after graft. Depending on positivity outcome, additional tests were performed at 1- to 2-week intervals		
Hiwarkar, 2013[58]	United Kingdom (Jun 2005-Dec 2010)	Retrospective	NR	PBSC, BM, UCB	278	Median: 33 months<br>Range: (0.5-197) months	Pediatrics	EBV load ≥40000 copies/mL	Twice weekly until recovery of the CD4 T-cell count >0.3 x 10^9 L^{-1}	Whole blood	Chi2 test with Yates correction was used to identify the potential risk factors for EBV. Variables with a p-value <0.2 in univariate analysis were considered in a multivariate logistic regression model
Hoegh-Petersen, 2011[59]	Canada (Jan 2004-Jan 2009)	Retrospective	Median: 375 days<br><br>Range: 28-1727 days	PBSC, BM	307<br>No PTLD: 282<br><br>PTLD:25	No PTLD:<br><br>Median:47 years<br>Range:18-66 years<br><br>PTLD:<br><br>Median:53 years<br>Range:20-65 years	Adults<br><br>All patients received ATG	Proven PTLD was defined as histologically diagnosed PTLD. Probable PTLD was defined as typical clinical manifestation(s) of PTLD (unexplained fever, lymphadenopathy, splenomegaly, lymphocytosis or imaging-diagnosed mass), with EBV DNAemia above 400 copies per µg blood DNA	NA	NA	Chi 2 test or Fisher's exact test for categorical variable, Mann-Whitney-Wilcoxon rank-sum test for continuous variables
Hoshino, 2001[60]	Japan (July 1998-july 2000)	Prospective	NR	BM, UCB, PBSC	38	Mean: 8.6 years<br>Range: (5 months -35 years)	Pediatrics & Adults	EBV load >$10^{2.5}$ copies/ µg DNA	Collection of blood samples started from the 2nd or 3rd week post-transplant. Tests were performed weekly if there were symptoms of lympho-proliferative syndrome. In the absence of symptoms by 3 months, routine follow-up was stopped.	Peripheral blood	Fisher's exact test
Islam, 2010[61]	United Kingdom (March 2001 to July 2008)	Retrospective	Median: 4.2 years<br>Range: (0.9-8.1) years	BM, PBSC, UCB	48 non-malignant subgroup and 35 malignant subgroup)	NR	Pediatrics & Adults	50 EBV genome copies/mL	Twice weekly until 3 months; once weekly until 6 months and thereafter once every 3 weeks until 12 months post-transplant;	Whole blood	Mann-Whitney, Chi2 test, Fisher's exact test Univariate logistic regression

Table S3: Characteristics of the 77 studies included in the systematic review

First author, year	Country (Date of graft)	Study type	Post-graft follow-up duration	Population: Graft type	Population: Sample	Population: Age	Population: Pediatrics /Adults	EBV DNAemia/PTLD definition	Frequency of testing	Blood compartment used for the test	Statistical methods
									additional when clinically indicated		
Issa, 2019[62]	USA (October 2007-September 2016)	Retrospective	NR	BM, PSBC	357	Median 57 years (range: 19-74 years)	Adults	EBV but not specified	Weekly through day +100 post-transplant	Serum	Gray's test
Jaskula, 2010[64]	Poland (2004-2009)	Prospective	1 year	Allogeneic-HSCT	102	≤16 years (16 persons, mean 6.5 years) >16 years (86 persons, mean 42 years)	Pediatrics & Adults	EBV DNA >100 copies/10^5 cells	The tests were performed weekly until the 30th post-transplant day. Then, monthly for 1 year as well as when there were clinical symptoms of reactivation. The number of measurements ranges between 3 and 30 (median, 9).	Peripheral blood	Fisher's exact test and Mann-Whitney test for univariate analysis. Logistic regression for multivariate analysis
Juvonen, 2007[65]	Finland (1988-1999)	Retrospective	>52 months (mainly in the first 3 months after transplant)	BM, PBSC	406	NR	Adults	EBV DNA levels >500 genome equivalents/mL	At least 1 sample of each patient's serum was collected weekly during hospitalization and during post-discharge visits. The median number of samples per patient was 14 (range: 1-26).	Serum	Multivariate Cox model
Kalra, 2018[66]	Canada (Jan 2007-Sept 2015)	Retrospective	Median: 509 days<br><br>Range: 6-2576 days	UCB, PBSC, BM	554<br>No PTLD: 500<br><br>PTLD:54	No PTLD:<br><br>Median:51 years<br>Range:16-67 years<br><br>PTLD:<br><br>Median:44.5 years<br>Range:18-66 years	Pediatrics & Adults<br><br>All patients received ATG	Between Jan 2007 and Apr 2012 PTLD was diagnosed by biopsy. Between May 2012 and Sept 2015 PTLD was diagnosed as at least one symptom/sign/radiologic evidence of PTLD plus EBV DNAemia >40 000 copies/ml. If fever was the only manifestation of PTLD, EBV DNAemia of >400 000 copies/ml was required for the diagnosis of PTLD.	NA	NA	Univariate and multivariate competing risk regression (Fine-Gray model)
Kullberg-Lindh, 2011[67]	Sweden (January 2001-December 2005)	Retrospective	6 months	BM, PBSC, CB	47	Median: 8.6 years<br>Range: (0.9-18) years	Pediatrics	Maximum viral DNAemia	EBV DNA was followed once a week from day 0 to day 100, and, based on clinical suspicion	Serum	Univariate and multiple linear regression
Kutnik, 2019[63]	Poland (2001-2018)	Retrospective	Median 12 months	PBSC, BM	198	Range: 0-18 years	Pediatrics	EBV but not specified	NR	NR	Chi 2 test

Table S3: Characteristics of the 77 studies included in the systematic review

First author, year	Country (Date of graft)	Study type	Post-graft follow-up duration	Population				EBV DNAemia/PTLD definition	Frequency of testing	Blood compartment used for the test	Statistical methods
				Graft type	Sample	Age	Pediatrics /Adults				
Laberko, 2017[68]	Russia (May 2012 to December 2014)	Retrospective	Median 27 months	PBSC	182	Median 6.4 years Range:0.2-23.0	Pediatrics & Adults	EBV above 500 copies viral DNA/mL	Weekly until day 100, thereafter tailored based on continuing immuno-suppression, previous history of viral reactivation, and immune reconstitution	Whole blood	2-sided log-rank test or Mann-Whitney test for univariate analysis. Fine and Gray competitive risk model, for multivariate analysis
Landgren, 2009[69]	CIBMTR, USA (1968-1994) FHCRC, Seattle (1969-1996)	Multi-institutional retrospective	>120 months Median> 12 months	Allo-BMT	271 transplant centers 26901	Median 26.6 years Range:0.1-68	Pediatrics & Adults	Of the 127 PTLD cases, 116 were confirmed by centralized histopathologic review of archived tissue or slides or by review of pathology/clinical reports. The information transferred by the transplant centers was considered for 11 cases.	NA	NA	Poisson regression methods for grouped survival data.
Li, 2018[70]	China (January 2006 to December 2016)	Retrospective	Median: 32.5 months Range (0.5- 132) months	BM, PBSC	62	Median: 7 years 1 months Range: (1 year 2 months to 16 years 9 months)	Pediatrics	Not provided	Not provided	Not provided	Chi2 test
Lin, 2019[71]	China (June 2013-January 2016)	Multicenter randomized study	1 year	PBSC, BM	408	Range: 14-59 years	Pediatrics & Adults	EBV-DNA in blood positive (≥500 copies/mL) twice consecutively	Weekly for the first 3 months after transplantation, once every 2 weeks from the 4th to the 9th month post-transplantation and then once per month from the 10th to the 12th month.	Plasma	Multivariate Cox model
Liu, 2020[72]	China (March 2016-March 2018)	Prospective	NR	PBSC, BM	170	Range: 18-60 years	Adults	EBV-DNA >1000 copies/mL on more than two consecutive occasions	Weekly until day 100 post-transplantation	Peripheral blood	Chi 2, Mann-Whitney U tests and Cox model
								Probable PTLD	NA	NA	Chi 2 and Mann-Whitney U tests
Liu, 2018[74]	China (February 2016 to August 2016)	Prospective	100 days	BM, PBSC	132	Range: (18-59) years	Adults	Two or more consecutive EBV-DNA tests at >1000 copies/mL	Weekly until day 100 after transplantation	Peripheral blood	Mann–Whitney U test, Chi2 test, time-dependent landmark study
Liu, 2013[73]	China (Feb 2009-Aug 2012)	Prospective	Median (range) : 327 (27-1408) days	PBSC, PBSC+ BM, BM	251[g]	Median: 28 years Range: (12-63) years	Pediatrics & Adults	≥500 genome copies/mL.	Weekly during the first 3 months. Biweekly between the 4th and 9th months. Monthly between the 10th and 24th months. Every 3 months between the 25th and 36th months.	Plasma	Univariate and multivariate Cox models. (Variables for multivariate models were selected using backward stepwise elimination with p>0.05 for removal)

Table S3: Characteristics of the 77 studies included in the systematic review

First author, year	Country (Date of graft)	Study type	Post-graft follow-up duration	Population				EBV DNAemia/PTLD definition	Frequency of testing	Blood compartment used for the test	Statistical methods
				Graft type	Sample	Age	Pediatrics /Adults				
									Once a positive result was obtained, follow-up testing was done twice weekly.		
Liu, 2013[26]	China (July 2008-May 2011)	Prospective	Median (range) : 495 (45-1158) days	PBSC, PBSC+ BM, BM	172	Median: 29.5 years Range: (12-61) years	Pediatrics & Adults	≥ 500 genome copies/mL	Weekly during the first 3 months. Biweekly between the 4th and 9th months. Monthly between the 10th and 24th months. Trimonthly between the 25th and 36th months. Once a positive result was obtained, follow-up testing was done twice weekly.	Plasma	Logrank test
								Proven PTLD	NA	NA	
Marinho-Dias, 2019[75]	Portugal (January 2015-December 2015)	Prospective	Median> 120 days	PBSC, BM, UCB	40	Median 32.2 years (range: 1-63 years)	Pediatrics & Adults	Positive EBV PCR test	Day (D)+30, D+60, D+90, D+120, D+150 and D+180 post-transplant	Whole blood	Chi 2 or Fisher's exact tests for univariate analysis and Cox model for multivariate analysis.
Meijer, 2004[76]	Netherlands (September 2001-December 2003)	Prospective	Nonmyelo-ablative group<br><br>Mean: 19 months Range: (7-32) months<br><br>Myeloabla-tive group<br><br>Mean: 14 months Range: (6-31) months	BM PBSC	78h<br><br>40 in nonmyeloablative group<br><br>38 in myeloablative group	Nonmyeloablative group Median: 56 years Range: (24-67) years<br><br>Myeloablative group Median: 44 years Range: (21-55) years	Adults	≥1000 copies/mL	Weekly until day 120 post-transplant. Thereafter monitoring was continued bi-weekly until day 180 for recipients of an myeloablative regimen, and until 1-year post-transplant for recipients of a non-myeloablative regimen.	Plasma	Wald test
Mountjoy, 2020[77]	USA (January 2007-December 2016)	Retrospective	Non-ATG group Median 677 days (range: 7-3147 days)<br><br>ATG group Median 504 days (33-2156 days)	HSCT	209	Non-ATG group Median: 53 years (range:18-74 years)<br><br>ATG group Median: 56 years (range:19-71 years)	Adults	Elevation in viral copy number	At least every 2 weeks until day 100 post-transplant and then at the discretion of the doctor depending on the immunosuppression and the clinical status of the patient.	Peripheral blood	Chi 2 test
Neumann, 2018[78]	Germany (2001 to 2012)	Case-control	2 years	Not provided	44	Median: 49.2 years Range: (19.8-70.0) years	Adults	Not provided	Not provided	Peripheral blood	Wilcoxon rank sum test

Table S3: Characteristics of the 77 studies included in the systematic review

First author, year	Country (Date of graft)	Study type	Post-graft follow-up duration	Population				EBV DNAemia/PTLD definition	Frequency of testing	Blood compartment used for the test	Statistical methods
				Graft type	Sample	Age	Pediatrics /Adults				
Nowak, 2019[79]	Poland (2002-2012)	Retrospective	Median 2.1 months (range 0.2-67.8 months)	PBSC, BM	239	Median 31.6 years (range: 1.0-61.5 years)	Pediatrics & Adults	EBV infection but not specified	NR	NR	Univariate Cox model
Omar, 2009[80]	Sweden (July 2005-June 2007)	Prospective	NR	BM, PBSC, CB	131	Median: 39 years Range:(0.3-70) years	Pediatrics & Adults	EBV viral load was analysed as a continuous variable. The interval of 50-500 copies/mL was set to 225 copies/mL and log_{10} values were used in analyses.	High-risk group: weekly during first 3 months. Standard risk group: no routine monitoring, only if clinical suspicion of EBV infection.	Serum	Multiple linear regression
Pagliuca, 2019[81]	France (2010-2017)	Retrospective	Median 47.33 months (range: 3.18-126.20 months)	BM, CB, PB	208	Median: 42.52 years (range: 8.35-74.77 years)	Pediatrics & Adults	Proven or probable EBV-PTLD	NA	NA	Fine and Gray model. Stepwise backward procedure was used. All predictors with a p <0.10 were considered and sequentially removed if the pvalue in the multivariable model was >0.05.
Park, 2020[82]	Korea (August 2004-April 2016)	Retrospective	NR	HSCT	114	Median 43.5 years (range: 2-71 years)	Pediatrics & Adults	EBV infection but not specified	NR	NR	Chi 2 or Fisher's exact tests
Patriarca, 2013[4]	Italy (Jan 2008-Dec 2010)	Prospective	Median: 7 months Range: (2-36) months	PBSC, BM	100[i]	Median: 50 years Range: (20-70) years	Adults	≥10000 genome copies/mL	Weekly during the first 3 months post-transplant and biweekly between the 3rd and 6th months.	Whole blood	Variables with a p-value ≤0.1 in univariate analysis were considered in a multivariate logistic model
Peric, 2012[83]	France (Jan 2005-Jun 2009)	Retrospective	Median: 468 days Range: (92-1277) days	UCBT	33	Median: 50 years Range: (18-66) years	Adults	EBV PCR load above 1000 copies EBV DNA/10^5 cells	The tests were performed weekly during the first 6 months post-transplant. After 6 months, if there was no reactivation, the tests were performed monthly and whenever clinically relevant.	Peripheral blood	Mann-Whitney test and Fisher's Exact test
Peric, 2011[84]	France (Jan 2005-Jun 2009)	Retrospective	Median: 655 days Range: (92-1542) days	PBSC, BM	175	Median: 56 years Range: (18-71) years	Adults	EBV PCR load above 1000 copies EBV DNA/10^5 cells	Weekly during the first 6 months post-transplant and thereafter when clinically relevant.	Peripheral blood	The variables with a p-value <0.3 in univariate analysis (Mann-Whitney test or Fisher's exact test) were considered in a Fine Gray model
Ru, 2020[85]	China (July 2011-July 2014)	Retrospective	NR	HSCT	890	Median 32 years (range: 2-63 years)	Pediatrics & Adults	EBV-VL≥10^2 copies/mL	Weekly until day 90 post-transplant and once every 2 weeks from +90 days until +180 days. After this date, tests were carried out in the	Whole peripheral blood	Univariate and multivariate Cox models. Only variables with pvalue <0.1 in univariate analyse were considered in

Table S3: Characteristics of the 77 studies included in the systematic review

First author, year	Country (Date of graft)	Study type	Post-graft follow-up duration	Population				EBV DNAemia/PTLD definition	Frequency of testing	Blood compartment used for the test	Statistical methods
				Graft type	Sample	Age	Pediatrics /Adults				
									presence of clinical signs		multivariate model.
Rustia, 2016[86]	United States of America (2008-2014)	Retrospective	180 days	CB, PBSC, BM	140	Mean: 9.46 years<br>SD: (6.09)<br>Range: (0.25-22) years	Pediatrics	≥1000 copies/mL on 2 consecutive PCR tests	Weekly for 180 days after transplant	NR	Chi2 test
Sanz, 2014[87]	Spain (May 1997 to December 2012)	Retrospective	3 years	UCB	288<br>-241 MAC group<br>- 47 RIC group	MAC group<br>Median: 34 years<br>Range: (16-57) years<br>RIC group<br>Median: 46 years<br>Range: (13-65) years	Pediatrics & Adults	EBV viremia detected on at least 2 consecutive samples (positivity cut-off: 900 copies of EBV DNA per mL of plasma)<br>Proven EBV-PTLD was defined according to the European Conference on Infections in Leukemia guidelines.	Weekly from day 7 post-transplant to day 100, every 2 weeks until day 180 and monthly thereafter for the first year.<br>NA	Plasma<br>NA	Gray test for comparisons cumulative incidence. Fine and Gray for competing events (relapse and death without EBV reactivation) used for multivariate analyses with variables with a p-value < 0.10.
Sirvent-von Bueltzingsloewen, 2002[88]	France (Oct 1995-May 1998)	Multicenter Prospective	Median: 306 days<br>Range: (26-867) days	SCT	85[j]	NR	Pediatrics & Adults	>300 copies/µg DNA	Prior to transplantation and on days +30, +60 and +90 post-transplantation	Peripheral blood	Chi-square test, and logistic regression for multivariate analysis
Styczynski, 2013[89]	EBMT (1999-2011)	Multicenter retrospective	NR	CB, PBSC, BM	19 transplant centers<br>4466	NR	Pediatrics & Adults	Proven PTLD was diagnosed by biopsy or other invasive procedure, with a test with appropriate sensitivity and specificity together with symptoms and signs from the affected organ. Probable PTLD was defined as significant lymphoadenopathy or other end-organ disease accompanied by a high EBV-DNA blood load, in the absence of other etiologic factors or established diseases	NA	NA	NR
Torre-Cisneros, 2004[90]	Spain (Oct 1999-Jan 2002)	Prospective	12 months	BMT	100[k]	Median: 22 years<br>Range: (5-50) years	Pediatrics & Adults	≥ 50 genome equivalents/mL plasma	Biweekly for the first 100 days and then monthly until the 12th month post-transplant. Further tests were carried out when clinically indicated.	Plasma	Univariate and Multivariate Cox models

Table S3: Characteristics of the 77 studies included in the systematic review

First author, year	Country (Date of graft)	Study type	Post-graft follow-up duration	Population				EBV DNAemia/PTLD definition	Frequency of testing	Blood compartment used for the test	Statistical methods
				Graft type	Sample	Age	Pediatrics /Adults				
Trottier, 2012[91]	Canada (1993-2009)	Retrospective	1 year	PBSC, UCB, BM	238	NR	Pediatrics	EBV-VL above minimum threshold value (value NR)	EBV-viral load was tested at regular intervals of two weeks or less for approximately 4 months or as long as immuno-suppression persisted (following hospital protocol)	NR	Multivariate Cox model
Tsoumakas, 2019[92]	Greece (September 2011-September 2015)	Prospective	≥1 year	BM, PBSC	110	Median: 8 years (range: 0.08-18.5 years)	Pediatrics	Positive EBV PCR test	Weekly until day 100. After that, tested whenever clinically suspected	Peripheral blood	Univariate and multivariate Cox models
Uhlin, 2014[93]	Sweden (1996-2011)	Retrospective	NR	PBSC, BM and UCB	1021	Patients without PTLD Median: 38 years Range: <1-77 years<br><br>Patients with PTLD Median: 37 years Range: 1-67 years	Pediatrics & Adults	PTLD was diagnosed according the histological criteria reported for B-cell lymphoproliferative states following transplantation.	NA	NA	Gray test for univariate analysis and Fine-Gray competitive risk model for multivariate analysis.<br>All factors with p≤0.1 in univariate analysis were included in the multivariate model. A stepwise backward procedure has used to retain in the model factors with p≤0.05.
van der Velden, 2013[94]	The Netherlands (2006-2011)	Retrospective	Minimum 6 months of follow-up	Allo-SCT	273 EBV infection (61) No EBV infection (212)	**EBV infection** Median: 47 years Range: (19-66) years **No EBV infection** Median: 51 years Range: (19-66) years	Adults	EBV-DNAemia was considered synonymous with infection when the PCR for EBV-DNA was ≥log 3 copies/ml. and EBV disease was defined as either probable EBV disease or proven disease	NA	NA	t-test, Mann–Whitney U-test or Fisher's exact test for the univariate analysis. Only variables with p≤0.2 on univariate analysis were considered in backward logistic regression analysis.
Van Esser, 2001[95]	Netherlands, Germany, Italy (March 1996-Jun 1999)	Multi-country Prospective	180 days	Allo-SCT	Total: 152 85 TCD-SCT and 67 non-TCD-SCT	TCD-SCT group Median: 41 years Range: (17-55) years<br><br>non-TCD-SCT group Median: 31 years Range: (17-56) years	Pediatrics & Adults	EBV DNA level in plasma exceeding 50 genomes equivalents/mL	Biweekly up to 180 days post-transplant.	Plasma	Variables with a p-value <0.05 in univariate analysis (Log-rank test and Cox model) were considered in a multivariate Cox model
								Proven PTLD	NA	NA	
Wang, 2019[96]	China (December 2007 - June 2016	Retrospective	Until death or 08 February 2017	BM, PBSC	186	Median 39 years (range: 7-62 years)	Pediatrics & Adults	EBV-VL≥1000 copies/mL	Every week for three months post-transplantation, and every two weeks between day 90 to day 180	Peripheral blood	Univariate and multivariate Fine and Gray models.

Table S3: Characteristics of the 77 studies included in the systematic review

First author, year	Country (Date of graft)	Study type	Post-graft follow-up duration	Population				EBV DNAemia/PTLD definition	Frequency of testing	Blood compartment used for the test	Statistical methods
				Graft type	Sample	Age	Pediatrics /Adults				
									post-transplantation		
Xu, 2015[97]	China (2006-2012)	Case-control study	NR	Haploidentical HSCT	PTLD: 45 Controls: 135 Each PTLD case was matched to 3 controls randomly selected from the same the cohort. Matching criteria included age at the time of HSCT (±5 years), time of the HSCT (±4 months), and transplantation duration (±3 months).	PTLD Median:25 years Range:3-49 years<br><br>Controls Median:27 years Range:3-48 years	Pediatrics & Adults	Proven PTLD was diagnosed if EBV were detected in a specimen obtained from an organ by biopsy or other invasive procedure according to a test with appropriate sensitivity and specificity together with symptoms and signs from the affected organ. Probable PTLD was defined as significant lymphoadenopathy or other end organ disease accompanied by a positive EBV-DNA blood load in the absence of other etiologic factors and established diseases.	NA	NA	Univariate and multivariate Cox regression. Factors with p-value <.10 in the univariate analysis were included in the multivariate regression
Xuan, 2012[98]	China (Feb 2009-Dec 2011)	Prospective	Median (range) : 319 (27-1194) days	PBSC, PBSC +BM, BM	185	Median: 28 years Range: (12-63) years	Pediatrics & Adults	≥500 genome copies/mL	Weekly during the first 3 months. Biweekly between the 4th and 9th months. Monthly between the 10th and 24th months. Trimonthly between the 25th and 36th months. Once a result was positive, the testing was done twice weekly.	Plasma	Univariate and multivariate Cox models (backward stepwise elimination with p≥ 0.05 for removal) Conditioning group variable was included regardless of significance.
Xuan, 2013[16]	China (July 2008 - June 2012)	Prospective	Median (range): 374 (27-1554) days	Allo-HSCT	263	Median: 29 years Range: (11-63) years	Pediatrics & Adults	EBV-PTLD was diagnosed according to the criteria of World Health Organization	NA	NA	Univariate and multivariate Cox regression models
Yu, 2019[99]	China (September 2016-March 2017)	Prospective	NR	Allo-HSCT	90 (45 for long-term MMF treatment group and 45 for short-term treatment group)	Long-term group Median 29 years (range: 15-58 years)<br><br>Short-term group Median 35 (range: 14-58 years)	Pediatrics & Adults	EBV infection but not specified	NR	NR	Univariate and multivariate Cox models. Only variables with p-value <0.1 in univariate analyse were considered in multivariate model. Using a forward stepwise approach, only variables with pvalue<0.05 were retained in final model.

Table S3: Characteristics of the 77 studies included in the systematic review

First author, year	Country (Date of graft)	Study type	Post-graft follow-up duration	Population				EBV DNAemia/PTLD definition	Frequency of testing	Blood compartment used for the test	Statistical methods
				Graft type	Sample	Age	Pediatrics /Adults				
Zallio, 2013[23]	Italy (March 2005-Dec 2011)	Prospective	180 days	PBSC, BM, UCB	100	Median: 50 years Range: (20-70) years	Adults	>500 genome copies/mL	Weekly during the first 3 months post-transplant. If GvHD after day 100, test was continued until immunosuppressive therapy discontinuation	Whole blood	Chi2 test for univariate analysis and Logistic regression model for multivariate analysis[l]
Zhou, 2020[100]	China (November 2008-June 2016)	Retrospective	Median 59.2 months (range: 2.03-113.8 months)	BM, PBSC	131	Median 18 years (range: 2-58 years)	Pediatrics & Adults	EBV DNA>500 copies/mL in two consecutive time points without EBV-associated disease.	Every week in the first 30 days post-transplant and every two weeks until 3 months post-transplant or until EBV DNA copies was undetectable.	Whole blood	Univariate and multivariate Cox models. Only variables with pvalue <0.1 in univariate analyse were considered in multivariate model.
Zhou, 2020[101]	China (November 2007-June 2015)	Retrospective	Median 64.7 months (range, 2.03-113.8 months),	Haplo-HSCT	116	Range: 4-58 years	Pediatrics & Adults	Probable and proven PTLD	NA	NA	Cumulative incidence method in presence of competing event

[a]Alemtuzumab has been considered in the conditioning protocol of all patients and only patients with at least 6 months of follow-up were considered.
[b]Almost all patients received the standard conditioning regimen.
[c]All of these patients had positive EBV serology, survived beyond 40 days and received cyclosporine beyond 30 days post-transplant.
[d]Of the 40 patients, 5 were excluded: 3 because of related early transplant mortality and 2 dues to relapse before 60 days of follow-up.
[e]Factors associated with EBV reactivation were assessed after 100 days of follow-up. It should be noted, however, that a follow-up period of 2 years was considered for the diagnosis of cases of post-transplantation lymphoproliferative syndrome.
[f]All patients in the study had positive CMV serology and negative PCR tests for herpes viruses (EBV, CMV, and HHV-6) one week after transplantation.
[g]All patients had a negative EBV PCR test at the start of follow-up.
[h]All except 1 (receiving bone marrow), received a peripheral blood stem cell graft.
[i]All patients had a follow-up duration > 30 days post-transplant.
[j]Five patients with post-transplant lymphoproliferative syndrome were excluded. Analysis of risk factors for EBV reactivation concerns 80 patients.
[k]All patients had positive EBV serology before transplantation.
[l]The information on the use of the logistic regression model does not appear in the article; it was given to us by the first author of the article.

Abbreviations:
Allo: allogeneic; **Allo-HSCT**: allogeneic hematopoietic stem cell transplantation; **BM**: bone marrow; **BMT**: bone marrow transplant; **CB**: cord blood; **CIBMTR**: Center for International Blood and Marrow Transplant Research; **CIC**: conventional-intensity conditioning; **DNA**: deoxyribonucleic acid; **EBV**: Epstein-Barr virus; ; **EBV-VL**: EBV viral load; **EMBT**: European Group for Blood and Marrow Transplantation; **FHCRC**: Fred Hutchinson Cancer Research Center; **GvHD**: graft-versus-host disease; **Haplo-HSCT**: haploidentical hematopoietic stem cell transplantation; **HSCT**: hematopoietic stem cell transplantation; **IBMTR**: International Bone Marrow Transplant Registry; **IQR**: interquartile range; **MAC**: myeloablative conditioning; **MMF**: mycophenolate mofetil; **NA**: not applicable; **NR**: not reported; **PBMC**: peripheral blood mononuclear cells; **PBSC**: peripheral blood stem cells; **PCR**: polymerase chain reaction; **PTLD**: post-transplant lymphoproliferative disorders; **RIC**: reduced-intensity conditioning; **SCT**: stem cell transplant; **SD**: standard deviation; **TCD**: T-cell depletion; **UCB**: umbilical cord blood; **UCBT**: umbilical cord blood transplant.

Table S4: Risk factors for post-transplant EBV infection and for PTLD explored in the 77 retained studies

First author, year	Outcome	Study population	Risk factors explored	Estimate (95% CI); p-value	
				Univariate results	Multivariate results
			**Recipient age**		
Bogunia-Kubik, 2007[34]	EBV	P & A	> vs. ≤ 25 years	NR	**OR=1.54 (1.136-2.703); p=0.034**
Burns, 2016[38]	EBV	P & A	≥ 50 years vs. < 50 years	**HR=1.54 (1.02-2.31); p=0.039**	HR=1.30 (0.76-2.23); P=0.342
Dumas, 2013[49]	EBV	P & A	>18 vs. ≤18 years	**p=0.008**	NS
Elmahdi, 2016[51]	EBV	P	≥10 vs. <10 years	HR=0.646 (0.261-1.741); p=0.39	
Tsoumakas, 2019[92]	EBV	P	≥ 8 vs <8 years	HR=1.22 (0.52-2.88)	NI
Liu, 2013[73]	EBV	P & A	< 20 vs. ≥20 - ≤40 vs. >40 years	NS	NS
Gao, 2019[55]	EBV	P & A	≥40 vs. <40 years	p=0.229	NI
Marinho-Dias, 2019[75]	EBV	P & A	≥20 vs. <20 years	OR=2.50 (0.62-10.1); p=0.173	-
Marinho-Dias, 2019[75]	EBV	P & A	≥35 vs. <35 years	OR=1.61 (0.41-6.34); p=0.366	-
Ru, 2020[85]	EBV	P & A	<30 vs. ≥30 years	**HR=1.218 (1.049-1.413); p=0.010**	HR=1.041 (0.763-1.420); p=0.799
Czyżewski, 2019[47]	EBV	P & A	Children vs. Adults	**OR=15.7 (9.2-26.1); p<0.0001**	-
Zhou, 2020[100]	EBV	P & A	≤18 vs. >18 years	HR=0.750 (0.307-1.835); p=0.529	NI
Sirvent-von Bueltzingsloewen, 2002[88]	EBV	P & A	< 18 vs. ≥18 years	p=0.12	NS
Carpenter, 2010[22]	EBV	P & A	Continuous	NR	HR=0.989 (0.9-1.01); p=0.318
Kullberg-Lindh, 2011[67]	EBV	P	Continuous	slope=-0.03; p=0.58	slope=-0.06; p=0.09
Düver, 2020[50]	EBV	P	Age (continuous)	-	OR=1.08 (1.00–1.17); p=0.057
Laberko, 2017[68]	EBV	P & A	Continuous		HR= 1.026 (0.97-1.08); p= 0.36
Peric, 2011[84]	EBV	A	Continuous	p=0.97	-
Patriarca, 2013[4]	EBV	A	Continuous	p=0.498	-
Van Esser, 2001[95]	EBV	P & A	Continuous	NS	
Jaskula, 2010[64]	EBV	P & A	Categories unspecified	NR	NR (NS)
Sanz, 2014[87]	EBV	P & A	Continuous	NR	NR (NS)
Auger, 2014[33]	EBV	A	Median age of patients with EBV reactivation vs. median age of patients without EBV reactivation	NS	-
Cesaro, 2010[41]	EBV	P	< vs. ≥8.97 (median) years	p=0.6	
Comoli, 2007[46]	EBV	P & A	Categories unspecified	NS (NR)	
Han, 2014[57]	EBV	P	≤10 vs. 11-20 years	p=0.857	-
Islam, 2010[61]	EBV	P & A	Median age of patients with EBV reactivation vs. median age of patients without EBV reactivation (Non-malignant group, Malignant group)	(p=0.20, p=0.23)	-
Peric, 2012[83]	EBV	A	Continuous	p=0.36	-
Ali, 2019[30]	PTLD	P	Age (continuous)	p=0.542	-
Gao, 2019[55]	PTLD	P & A	≥40 vs. <40 years	p=0.115	**HR=0.4 (0.2-0.9); p=0.032**
Landgren, 2009[69]	PTLD	P & A	≥50 years	-	**RR=5.1 (2.8-8.7)**
Liu, 2013[26]	PTLD	P & A	< 20 yrs vs. ≥20 - ≤40 yrs vs. >40 yrs	0.185	0.444
Kalra, 2018[66]	PTLD	P & A	≤45 vs. >45	SHR=1.67; p=0.05	SHR=1.09, p=0.79
Xu, 2015[97]	PTLD	P & A	≥18 vs. <18 years	HR=0.62 (0.29-1.30); p=0.205	
Xuan, 2013[16]	PTLD	P & A	<20 vs. ≥20 & ≤40 vs. > 40 years	NS	NS
Buyck, 2009[39]	PTLD	P & A	Continuous	HR=1.05 (0.99-1.12); p=0.12	-
Sanz, 2014[87]	PTLD	P & A	Continuous	NR	NR (NS)
Uhlin, 2014[93]	PTLD	P & A	Categories unspecified	NR	NR (NS)
Van der Velden, 2013[94]	PTLD	A	Categories unspecified	-	NR (NS)
			**Donor age**		
Bogunia-Kubik, 2007[34]	EBV	P & A	Categories unspecified	NR	NR (NS)
Lin, 2019[71]	EBV	P & A	≥27 vs. <27 years		HR=0.90 (0.63-1.29); p=0.570
Gao, 2019[55]	EBV	P & A	≥40 vs. <40 years	p=0.510	NI
Tsoumakas, 2019[92]	EBV	P	≥31.7 vs. <31.7 years	**HR=5.35 (1.8-15.92); p=0.003**	NI
Gao, 2019[55]	PTLD	P & A	≥40 vs. <40 years	p=0.792	NI
Kalra, 2018[66]	PTLD	P & A	≤45 vs. >45	SHR=2.09, p=0.03	SHR=1.93, p=0.10
Xu, 2015[97]	PTLD	P & A	≥ vs. < Median	HR=1.55 (0.77-3.15); p=0.224	
			**Recipient sex**		
Burns, 2016[38]	EBV	P & A	Male vs. female	HR=1.22 (0.80-1.88); p=0.360	-
Bogunia-Kubik, 2007[34]	EBV	P & A	Categories unspecified	NR	NR (NS)
Dumas, 2013[49]	EBV	P & A	Categories unspecified	p>0.15	-
Elmahdi, 2016[51]	EBV	P	Male vs. Female	HR=0.70 (0.27-1.84); p=0.472	
Jaskula, 2010[64]	EBV	P & A	Female vs. Male	p=0.07	OR=2.48; p=0.070
Kullberg-Lindh, 2011[67]	EBV	P	Male vs. Female	slope=-0.20; p=0.69	slope=-0.08; p=0.86

Table S4: Risk factors for post-transplant EBV infection and for PTLD explored in the 77 retained studies

First author, year	Outcome	Study population	Risk factors explored	Estimate (95% CI); p-value	
				Univariate results	Multivariate results
Laberko, 2017[68]	EBV	P & A	Male vs. Female	p=0.55	HR= 0.97 (.51-1.85); p=0.92
Liu, 2013[73]	EBV	P & A	Male vs. Female	NS	NS
Patriarca, 2013[4]	EBV	A	Categories unspecified	p=0.277	-
Peric, 2011[84]	EBV	A	Categories unspecified	p=0.85	-
Sanz, 2014[87]	EBV	P & A	Female vs. Male	NR	NR (NS)
Sirvent-von Bueltzingsloewen, 2002[88]	EBV	P & A	Categories unspecified	p=0.2	NS
Van Esser, 2001[95]	EBV	P & A	Categories unspecified	NS	-
Gao, 2019[55]	EBV	P & A	Female vs. Male	p=0.512	NI
Lin, 2019[71]	EBV	P & A	Female vs. Male		HR=0.70 (0.47-1.05); p=0.084
Marinho-Dias, 2019[75]	EBV	P & A	Female vs. Male	**OR=8.33 (0.93-100); p=0.033**	-
Marinho-Dias, 2019[75]	EBV	P & A	Female vs. Male	NR (at day +150 post-transplantation)	NS (NR)
Ru, 2020[85]	EBV	P & A	Male vs. Female	HR= 1.016 (0.847-1.181); p 0.835	NI
Zhou, 2020[100]	EBV	P & A	Female vs. Male	HR=1.240 (0.506-3.085); p=0.628	NI
Auger, 2014[33]	EBV	A	Male vs. Female	NS	-
Peric, 2012[83]	EBV	A	Male vs. Female	p=1.00	-
Cesaro, 2010[41]	EBV	P	Male vs. Female	p=0.8	
Chiereghin, 2016[42]	EBV	P	Male vs. Female	p=0.190	-
Comoli, 2007[46]	EBV	P & A	Categories unspecified	NS (NR)	-
Islam, 2010[61]	EBV	P & A	Male vs. Female (Non-malignant group, Malignant group)	(p=0.82, p=0.18	-
Gao, 2019[55]	PTLD	P & A	Female vs. Male	p=0.746	-
Buyck, 2009[39]	PTLD	P & A	Female vs. Male	HR=0.93 (0.16-5.57); p=0.94	-
Liu, 2013[26]	PTLD	P & A	Male vs. Female	p=0.333	p=0.276
Xu, 2015[97]	PTLD	P & A	Male vs. Female	HR=0.79 (0.36-1.75) p=0.562	-
Xuan, 2013[16]	PTLD	P & A	Male vs. Female	NS	NS
Sanz, 2014[87]	PTLD	P & A	Female vs. Male	NR	NR (NS)
Uhlin, 2014[93]	PTLD	P & A	Male/Female	NR	NR (NS)
Van der Velden, 2013[94]	PTLD	A	Categories unspecified	-	NR (NS)
			**Donor sex**		
Fan, 2016[52]	EBV	P & A	Male donor	NR	**OR=13.240 (2.0-87.39); p=0.007**
Jaskula, 2010[64]	EBV	P & A	Female donor	**p=0.03**	**OR=2.82; p=0.044**
Gao, 2019[55]	EBV	P & A	Female vs. Male	p=0.002	**HR=0.6 (0.4-1.0); p=0.034**
Zhou, 2020[100]	EBV	P & A	Female vs. Male	HR=0.481 (0.195-1.184); p=0.111	NI
Peric, 2011[84]	EBV	A	Categories unspecified	p=0.85	-
Bogunia-Kubik, 2007[34]	EBV	P & A	Categories unspecified	NR	NR (NS)
Gao, 2019[55]	PTLD	P & A	Female vs. Male	p=0.201	HR=0.9 (0.4-2.4); p=0.870
			**Donor/recipient sex**		
Cesaro, 2004[40]	EBV	P	Sex D/R (Female/male vs. Other)	p=0.8	-
Kutnik, 2019[63]	EBV	P	D/R Sex (Female-Female vs. Female-Male vs. Male-Female vs. Male-Male)	p=0.29	-
Garcia-Cadenas, 2015[56]	EBV	A	Female donor to male recipient	p=0.09	NS
Pagliuca, 2019[81]	PTLD	P & A	Sex mismatched (Yes vs. No)	-	**SHR=4.69 (1.35-16.22); p=0.015**
Garcia-Cadenas, 2015[56]	PTLD	A	Female donor to male recipient	p=0.9	-
Xu, 2015[97]	PTLD	P & A	Female donor to male recipient	HR=0.65 (0.26-1.64) p=0.365	-
Uhlin, 2014[93]	PTLD	P & A	Female donor to male	NR	NR (NS)
Bogunia-Kubik, 2007[34]	EBV	P & A	Categories unspecified	NR	NR (NS)
Jaskula, 2010[64]	EBV	P & A	Categories unspecified	NR	NR (NS)
Fan, 2016[52]	EBV	P & A	Categories unspecified	NR	NR (NS)
			**Diagnosis**		
Patriarca, 2013[4]	EBV	A	AL vs. (Lymphoma, MM, and others)	p=0.844	-
Peric, 2011[84]	EBV	A	Lymphoid vs. Myeloid malignancies	p=0.14	SHR=1.3 (0.4-1.5); p=0.72
Burns, 2016[38]	EBV	P & A	NHL vs. AML/MDS	**HR=0.10 (0.03-0.33); p=0.0001**	**HR=0.18 (0.05-0.57); P=0.004**
Burns, 2016[38]	EBV	P & A	ALL vs. AML/MDS	HR=0.80 (0.41-1.56); p=0.513	HR=0.89 (0.45-1.75); P=0.734
Burns, 2016[38]	EBV	P & A	HL vs. AML/MDS	HR=0.80 (0.34-1.84); p=0.585	HR=1.63 (0.64-4.16); P=0.308
Burns, 2016[38]	EBV	P & A	CLL vs. AML/MDS	HR=1.01 (0.48-2.11); p=0.989	HR=0.87 (0.41-1.85); P=0.724
Burns, 2016[38]	EBV	P & A	MPD vs. AML/MDS	HR=0.95 (0.43-2.10); p=0.905	HR=0.95 (0.43-2.11); P=0.907

Table S4: Risk factors for post-transplant EBV infection and for PTLD explored in the 77 retained studies

First author, year	Outcome	Study population	Risk factors explored	Estimate (95% CI); p-value	
				Univariate results	Multivariate results
Burns, 2016[38]	EBV	P & A	Other vs. AML/MDS	HR=1.26 (0.54-2.93); p=0.591	HR=3.01 (0.94-9.65); P=0.063
Carpenter, 2010[22]	EBV	P & A	HL vs. AML	NR	**HR=3.534 (1.514-8.249); p=0.004**
Carpenter, 2010[22]	EBV	P & A	NHL vs. AML	NR	HR=0.678 (0.249-1.848); p=0.448
Carpenter, 2010[22]	EBV	P & A	MPD vs. AML	NR	HR=2.006 (0.828-4.858); p=0.123
Carpenter, 2010[22]	EBV	P & A	CLL vs. AML	NR	**HR=3.767 (1.375-10.322); p=0.01**
Carpenter, 2010[22]	EBV	P & A	Other disease vs. AML	NR	HR=1.449 (0.486-4.319); p=0.506
Sanz, 2014[87]	EBV	P & A	Hodgkin's disease vs. other diagnosis	NR	**SHR=11.6 (3.4-40.0); P<0.0001**
Laberko, 2017[68]	EBV	P & A	Malignant vs. Non-malignant	p=0.49	HR= 1.19 (0.59-2.41); p=0.63
Hiwarkar, 2013[58]	EBV	P	hematological vs. primary immunodeficiency vs. metabolic	p>0.2	-
Gao, 2019[55]	EBV	P & A	Lymphoid malignancies vs. Myeloid malignancies	p=0.526	NI
Ru, 2020[85]	EBV	P & A	Lymphoma vs. Other	HR=1.218 (0.692-2.143); p=0.494	NI
Zhou, 2020[100]	EBV	P & A	Underlying disease (MDS vs. AL)	HR=1.705 (0.372-7.872); p=0.492	NI
Zhou, 2020[100]	EBV	P & A	Underlying disease (AA vs. AL)	HR=3.411 (0.932-12.475); p=0.064	HR=4.369 (0.484-39.451); p=0.189
Cohen, 2005[45]	EBV	P	PID vs. Not PID	**OR=2.53 (1.07-5.97)**	OR=1.19 (0.45-3.12)
Bogunia-Kubik, 2007[34]	EBV	P & A	Categories unspecified	NR	NR (NS)
Cesaro, 2010[41]	EBV	P	Non-malignant vs. Malignant	p=1.0	
Chiereghin, 2016[42]	EBV	P	Acute lymphoblastic leukemia vs. Severe aplastic anemia vs. Acute myeloid leukemia vs. Other	**p=0.027**	-
Chiereghin, 2019[43]	EBV	P & A	ALL vs. AML vs. CML vs. Other	p=0.924	-
Peric, 2012[83]	EBV	A	Myeloid malignancies vs. Lymphoid malignancies vs. Aplastic	p=0.28	-
Auger, 2014[33]	EBV	A	Aplastic anemia vs. Chronic myeloid leukemia vs. Acute leukemia and Myelodysplastic syndromes	NS	-
Auger, 2014[33]	EBV	A	Lymphoproliferative disorders (Lymphoma vs. Chronic lymphocytic leukemia vs. Myeloma)	NS	-
Comoli, 2007[46]	EBV	P & A	Categories unspecified	NS (NR)	-
Cohen, 2005[45]	PTLD	P	PID vs Not PID	OR=2.69 (0.72-10.1)	
Fujimoto, 2019[54]	PTLD	P & A	ALL vs. AML/MDS	HR=0.99 (0.69-1.44); p=0.98	HR=1.08 (0.75-1.57); p=0.68
			CML/MPD vs. AML/MDS	HR=0.94 (0.56-1.57); p=0.81	HR=1.55 (0.89-2.69); p=0.12
			Lymphoid malignancies vs. AML/MDS	HR=1.24 (0.88-1.75); p=0.22	HR=1.33 (0.92-1.92); p=0.13
			AA vs. AML/MDS	**HR=4.95 (3.47-7.07); p<0.001**	**HR=5.19 (3.32-8.11); p<0.001**
			Others vs. AML/MDS	HR=1.91 (0.97-3.76); p=0.06	HR=1.94 (0.97-3.89); p=0.06
Gao, 2019[55]	PTLD	P & A	Lymphoid malignancies vs. Myeloid malignancies	p=0.509	NI
Ali, 2019[30]	PTLD	P	ALL	**p=0.022**	**-**
			AML/MDS		
			SAA		
			Thalassemia		
			Metabolic disease		
			Other benign diseases		
			Other malignant diseases		
Althubaiti, 2019[31]	PTLD	P	Malignant vs. Non-malignant	p=0.616	-
Xu, 2015[97]	PTLD	P & A	Acute leukemia vs. Non acute leukemia	HR=0.93 (0.64-1.35) p=0.710	
Sanz, 2014[87]	PTLD	P & A	Categories unspecified	NR	NR (NS)
Uhlin, 2014[93]	PTLD	P & A	Lymphoma vs. Other	NR	NR (NS)
Uhlin, 2014[93]	PTLD	P & A	Lymphoid vs. Myeloid	NR	NR (NS)
Uhlin, 2014[93]	PTLD	P & A	Malignant vs. Non-malignant	NR	NR (NS)
Van der Velden, 2013[94]	PTLD	A	Categories unspecified	-	NR (NS)
			**Genotype**		
Bogunia-Kubik, 2005[35]	EBV	P & A	Recipient having Interferon-γ gene (IFNG) 3/3 genotype vs. other IFNG genotype	**p<0.001**	**OR=7.284; p=0.005**

Table S4: Risk factors for post-transplant EBV infection and for PTLD explored in the 77 retained studies

First author, year	Outcome	Study population	Risk factors explored	Estimate (95% CI); p-value	
				Univariate results	Multivariate results
Bogunia-Kubik, 2007[34]	EBV	P & A	Presence of the C-C chemokine receptor 5 (CCR5) deletion mutation	**p=0.008**	**OR=0.17 (0.034-0.803); p=0.026**
Nowak, 2019[79]	EBV	P & A	Presence of inhibitory KIR:HLA (Yes vs. No)	**HR=7.79 (1.88-32.32); p=0.0047**	-
Nowak, 2019[79]	EBV	P & A	Presence of activing KIR:HLA (Yes vs. No)	**HR=0.24 (0.07-0.79); p=0.019**	-
Wang, 2019[96]	EBV	P & A	Karyotype (Good vs. Int vs. Poor)	p=0.233	NI
Pagliuca, 2019[81]	PTLD	P & A	Presence of HLA DRB1*11:01 (Yes vs. No)	-	**SHR=4.85 (1.57-14.97); p=0.006**
			**Recipient, donor EBV, CMV serostatus**		
Bordon, 2012[36]	EBV	P	EBV (R+ vs. R-)		NS
Dumas, 2013[49]	EBV	P & A	EBV (R+ vs. R-)	p>0.15	-
Sirvent-von Bueltzingsloewen, 2002[88]	EBV	P & A	EBV serostatus (R+ vs. R-)	p=0.15	NS
Sanz, 2014[87]	EBV	P & A	EBV serostatus (R+ vs. R-)	NR	NR (NS)
Düver, 2020[50]	EBV	P	EBV serostatus (R+ vs. R- vs. Unknown)	p=0.37	NI
Cesaro, 2010[41]	EBV	P	EBV serostatus (R- vs. R+)	p=0.5	
Chiereghin, 2016[42]	EBV	P	EBV serostatus (R+ vs. R-)	p=0.133	-
Düver, 2020[50]	EBV	P	Donor EBV serostatus (D+ vs. D- vs. Unknown)	**p=0.032**	NS (NR)
Lin, 2019[71]	EBV	P & A	D/R EBV serostatus (D-/R+ vs. Other)		**HR=1.58 (1.01-2.46); p=0.046**
Laberko, 2017[68]	EBV	P & A	EBV serostatus D+/R- vs. D+/R+	p=0.3	**HR= 2.85 (1.12-7.28); p= 0.028**
Laberko, 2017[68]	EBV	P & A	EBV serostatus D-/R+ vs. D+/R+	p=0.26	HR= 0.32 (0.05-2.0); p= 0.22
Laberko, 2017[68]	EBV	P & A	EBV serostatus D-/R- vs. D+/R+	No events	No events
Laberko, 2017[68]	EBV	P & A	EBV serostatus Unknown vs. D+/R+	p=0.97	HR= 1.23 (0.53-2.9); p= 0.63
Liu, 2013[73]	EBV	P & A	D/R EBV serostatus Matches vs. Mismatches	NS	NS
Cesaro, 2004[40]	EBV	P	EBV (D-/R- vs. Other)	p=0.08	-
Zhou, 2020[100]	EBV	P & A	EBV serostatus (D+/R+ vs. D+/R−)	HR=3.316 (0.388-28.316); p=0.273	NI
Dumas, 2013[49]	EBV	P & A	Mismatch of EBV recipient serological status with maternal serology	p>0.15	-
Peric, 2011[84]	EBV	A	EBV serostatus R+ and D+ vs. R- and D- vs. R- and D+	p=1.00	-
Bogunia-Kubik, 2007[34]	EBV	P & A	D/R EBV serostatus (Categories unspecified)	NR	NR (NS)
Jaskula, 2010[64]	EBV	P & A	D/R IgG serostatus (Categories unspecified)	NR	NR (NS)
Cesaro, 2010[41]	EBV	P	EBV serostatus (D- vs. D+)	p=0.1	
Comoli, 2007[46]	EBV	P & A	D/R EBV serostatus NR	NS (NR)	-
Islam, 2010[61]	EBV	P & A	EBV IgG D- vs D+ (Non-malignant group, Malignant group)	(p=0.4, p=1)	-
Islam, 2010[61]	EBV	P & A	EBV IgG R- vs R+ (Non-malignant group, Malignant group)	(p=0.14, p=0.60)	-
Jaskula, 2010[64]	EBV	P & A	Presence of EBV IgG antibodies in the donor (Yes vs. No)	**p=0.03**	NS (NR)
Sanz, 2014[87]	PTLD	P & A	EBV serostatus (R+ vs. R-)	NR	NR (NS)
Uhlin, 2014[93]	PTLD	P & A	D EBV+ R EBV- vs. Others	NR	**SHR=4.97 (2.30-10.7); p<0.001**
Kalra, 2018[66]	PTLD	P & A	EBV serostatus D+R− vs D+R+	**SHR=2.96, p=0.03**	
Kalra, 2018[66]	PTLD	P & A	EBV serostatus D−R+ vs D+R+	SHR=1.36 p=0.47	
Kalra, 2018[66]	PTLD	P & A	EBV serostatus D+R− vs D-R+	SHR=2.09 p=0.24	
Xu, 2015[97]	PTLD	P & A	EBV serostatus (D+/R- vs. others)	HR=1.00 (0.58-1.71) p=1.000	
Xuan, 2013[16]	PTLD	P & A	D/R EBV serostatus matched vs. mismatched	NS	NS
Van der Velden, 2013[94]	PTLD	A	EBV serostatus (D+ or R+, R-/D+)	-	NR (NS)
Dumas, 2013[49]	EBV	P & A	CMV serostatus R+ vs. R-	p>0.15	-
Sanz, 2014[87]	EBV	P & A	CMV serostatus (R+ vs. R-)	NR	NR (NS)
Xuan, 2012[98]	EBV	P & A	D/R CMV serostatus matched vs. mismatched	NS	NS
Cesaro, 2004[40]	EBV	P	CMV serostatus (D-/R- vs. Other)	p=0.4	-
Peric, 2011[84]	EBV	A	CMV serostatus (R+ or D+ vs. R- and D-)	p=0.84	-
Peric, 2012[83]	EBV	A	CMV serostatus (R+ vs. R-)	p=1.00	-
Cesaro, 2010[41]	EBV	P	CMV serostatus (R- vs. R+)	p=1.0	
Cesaro, 2010[41]	EBV	P	CMV serostatus (D- vs. D+)	p=0.6	
Comoli, 2007[46]	EBV	P & A	Categories unspecified	NS (NR)	-
Buyck, 2009[39]	PTLD	P & A	CMV serostatus (R+ vs. R-)	HR=0.25 (0.03-2.20); p=0.21	-
Brunstein, 2006[37]	EBV/PTLD	P & A	CMV serostatus (R- vs. R+)	-	HR=3.0 (0.9-9.7) p=0.07
Sanz, 2014[87]	PTLD	P & A	CMV serostatus (R+ vs. R-)	NR	NR (NS)
			**CMV reactivation/infection**		
Bordon, 2012[36]	EBV	P	CMV viremia (Yes vs. No)		NS
Carpenter, 2010[22]	EBV	P & A	CMV reactivation (Yes vs. No)	NR	HR=0.89 (0.50-1.59); p=0.690

Table S4: Risk factors for post-transplant EBV infection and for PTLD explored in the 77 retained studies

First author, year	Outcome	Study population	Risk factors explored	Estimate (95% CI); p-value	
				Univariate results	Multivariate results
Zallio, 2013[23]	EBV	A	CMV reactivation (Yes vs. No)	**p=0.013**	**Significant but NR**
Chiereghin, 2016[42]	EBV	P	CMV infection (Yes vs. No)	p=0.690	-
Garcia-Cadenas, 2015[56]	EBV	A	CMV reactivation (Yes vs. No)ǂ	p=0.22	-
Patriarca, 2013[4]	EBV	A	CMV reactivation (Yes vs. No)	p=0.369	-
Gao, 2019[55]	EBV	P & A	CMV DNAemia (Yes vs. No)	**p<0.001**	**HR=5.9 (2.5-13.9); p<0.001**
Zhou, 2020[100]	EBV	P & A	CMV DNAemia (Yes vs. No)	**HR=84.00 (10.159-694.585); p=0.000**	**HR=97.754 (9.477-1008.304); p=0.000**
Chiereghin, 2019[43]	EBV	P & A	CMV infection (Yes vs No)	p=0.492	-
Torre-Cisneros, 2004[90]	EBV	P & A	Replication of CMV (Yes vs. No)	**HR=3 (1.5-6); p=0.0013**	HR=2 (0.7-7.1); p=0.12
Torre-Cisneros, 2004[90]	EBV	P & A	CMV load >2500 copies/mL	**HR=3 (1.7-6); p=0.0004**	HR=2.1 (0.9-7); p=0.061
Torre-Cisneros, 2004[90]	EBV	P & A	CMV disease	HR=1.3 (0.6-2.8); p=0.53	NI
Hiwarkar, 2013[58]	EBV	P	Positive donor and recipient serology (CMV or EBV) or host adenoviral infection	**OR=4.6; p<0.0001**	**Significant but NR**
Garcia-Cadenas, 2015[56]	PTLD	A	CMV reactivation (Yes vs. No)	p=0.1	NS
Gao, 2019[55]	PTLD	P & A	CMV DNAemia (Yes vs. No)	**p<0.001**	**HR=11.6 (1.2-114.4); p=0.036**
Xu, 2015[97]	PTLD	P & A	CMV DNAemia (Yes vs. No)	HR=6.12 (1.26-29.64); =0.024	**HR=5.68 (1.17-27.57); p=0.031**
			**Donor type**		
Burns, 2016[38]	EBV	P & A	Sibling vs. unrelated	HR=1.24 (0.83-1.87); p=0.291	-
Bogunia-Kubik, 2007[34]	EBV	P & A	(Sibling, family haploidentical/matched unrelated)	NR	NR (NS)
Juvonen, 2007[65]	EBV	A	Unrelated vs. Sibling	**p<0.0001**	HR=0.96 (0.41-2.26); P=0.93
Cesaro, 2004[40]	EBV	P	Familial vs. Unrelated	**p=0.01**	NS
Jaskula, 2010[64]	EBV	P & A	(Sibling, matched unrelated)	NR	NR (NS)
Cohen, 2005[45]	EBV	P	Unrelated vs. Related	OR=1.36 (0.59-3.15)	-
Düver, 2020[50]	EBV	P	Unrelated donor vs. Related donor	**p<0.001**	**OR=5.05 (1.24–20.63); p=0.024**
Tsoumakas, 2019[92]	EBV	P	Related donor vs. Unrelated donor	**HR=0.37 (0.15-0.96); p=0.042**	**HR=0.38 (0.15-0.98); p=0.045**
Marinho-Dias, 2019[75]	EBV	P & A	Unrelated donor (Yes vs. No)	**OR=8.0; p=0.043** at day (D) +150 post-transplant	**HR=8.8, p=0.030** at D+150
Patriarca, 2013[4]	EBV	A	Unrelated vs. Related	**p=0.016**	NS
Carpenter, 2010[22]	EBV	P & A	MMRD vs. MRD	NR	HR=0.601 (0.081-4.459); p=0.618
Carpenter, 2010[22]	EBV	P & A	MUD vs. MRD	NR	HR=0.843 (0.431-1.649); p=0.619
Carpenter, 2010[22]	EBV	P & A	MMUD vs. MRD	NR	HR=0.866 (0.387-1.942); p=0.727
Christopeit, 2013[44]	EBV	A	MRD vs. MUD vs. MMUD		OR=4.00 (0.37-43.14); p=0.253
Van Esser, 2001[95]	EBV	P & A	Sibling vs. Unrelated	**HR=1.8 (1.1-2.9); p=0.02**	HR=0.9 (0.3-2.9); p=0.8
Liu, 2013[73]	EBV	P & A	Related vs. unrelated	**p<0.001**	NS
Zallio, 2013[23]	EBV	A	MUD vs. MMUD vs. Sibling	**p=0.032**	NS
Peric, 2011[84]	EBV	A	MUD vs. MRD	p=0.19	SHR=1.59 (0.8-3.3)
Peric, 2011[84]	EBV	A	MMUD vs. MRD		SHR=2.72 (0.8-8.7)
Laberko, 2017[68]	EBV	P & A	Matched unrelated vs. Haploidentical	p=0.76	HR= 1.13 (0.60-2.10); p=0.71
Omar, 2009[80]	EBV	P & A	Unrelated + family mismatched donor vs. HLA-matched donor	NR	**p=0.04**
Chiereghin, 2019[43]	EBV	P & A	Related donor vs. Unrelated donor	p=0.406	-
Burns, 2016[38]	EBV	P & A	HLA mismatches ≥ 1 Ag vs. None	HR=0.93 (0.55-1.57); p=0.794	-
Cohen, 2005[45]	EBV	P	HLA-mismatch vs. HLA-match	OR=1.74 (0.74-4.12)	-
Dumas, 2013[49]	EBV	P & A	HLA disparity 6 of 6 vs. 5 of 6 vs. 4 of 6 vs. ≤ 3 of 6	p>0.15	
Elmahdi, 2016[51]	EBV	P	HLA mismatch 2-3 vs. 0-1	HR=1.74 (0.667-4.249); p=0.256	
Hiwarkar, 2013[58]	EBV	P	≥ 1 HLA Ag mismatch	**OR=2.2; p<0.05**	NS
Sirvent-von Bueltzingsloewen, 2002[88]	EBV	P & A	HLA non-genoidentical vs. HLA genoidentical	**p<0.01**	**OR=5 (1.5-16.4)**
Torre-Cisneros, 2004[90]	EBV	P & A	No HLA-matched sibling donor	**HR=2.8 (1.5-5.3); p=0.0014**	HR=2.1 (0.8-6.2); p=0.069
Patriarca, 2013[4]	EBV	A	HLA-mismatched vs. HLA-matched	**p=0.006**	NS
Liu, 2013[73]	EBV	P & A	HLA Matched vs. mismatched	**p<0.001**	NS
Jaskula, 2010[64]	EBV	P & A	HLA mismatched (Categories unspecified)	NR	NR (NS)
Fan, 2016[52]	EBV	P & A	HLA mismatched	NR	NR (NS)
Sanz, 2014[87]	EBV	P & A	HLA compatibility (6 of 6, 5 of 6, 4 of 6)	NR	NR (NS)
Peric, 2012[83]	EBV	A	HLA matching 5/6 vs. 4/6	p=0.18	

Table S4: Risk factors for post-transplant EBV infection and for PTLD explored in the 77 retained studies

First author, year	Outcome	Study population	Risk factors explored	Estimate (95% CI); p-value	
				Univariate results	Multivariate results
Gao, 2019[55]	EBV	P & A	Haploidentical donors vs. Matched sibling donors	**p<0.001**	HR=2.0 (0.8-5.1); p=0.130
Ru, 2020[85]	EBV	P & A	HLA-haploidentical vs. HLA-identical	**HR=2.670 (1.984–3.594); p<0.001**	**HR=1.830 (1.275-2.627); p=0.001**
Tsoumakas, 2019[92]	EBV	P	Matched graft vs. Mismatched graft	**HR=0.51 (0.21-1.22)**	NI
Cesaro, 2010[41]	EBV	P	Full A, B, DR matched vs. At least 1 allele or antigen mismatched	p=0.4	
Hoshino, 2001[60]	EBV	P & A	HLA Matched vs. Mismatched	p=0.627	-
Hoshino, 2001[60]	EBV	P & A	HLA-matched sibling vs. Alternative donor	p=0.559	-
Islam, 2010[61]	EBV	P & A	HLAIDSIB vs. MUD (Non-malignant group, Malignant group)	(p=1, p=0.39)	-
Atay, 2018[32]	EBV	P	MRD vs. 10/10 HLA allele-MUD vs. 9/10 HLA allele-MUD vs. HLA-haploidentical	p=0.25	
Auger, 2014[33]	EBV	A	Unrelated vs. Related vs. Cord blood	NS	-
Chiereghin, 2016[42]	EBV	P	Matched unrelated vs. Related	**p=0.039**	-
Li, 2018[70]	EBV	P	Haploidentical donor vs. MRD/MUD	p=0.11	
Althubaiti, 2019[31]	PTLD	P	Related donor vs. Unrelated donor	p=1.00	-
Ali, 2019[30]	PTLD	P	MRD vs. MMRD vs. MUD vs. MMURD	p=0.446	-
Pagliuca, 2019[81]	PTLD	P & A	Unrelated (Yes vs. No)	-	SHR=2.11 (1.00-4.45); p=0.051
Cohen, 2005[45]	PTLD	P	Donor unrelated vs. related	OR=2.22 (0.55-8.99)	
Liu, 2013[26]	PTLD	P & A	Donor related vs. unrelated	**p=0.001**	p=0.112
Xuan, 2013[16]	PTLD	P & A	Donor related vs. unrelated	p<0.001	NS
Buyck, 2009[39]	PTLD	P & A	Matched unrelated donor vs. HLA identical sibling	HR=2.26 (0.38-13.51); p=0.37	-
Kalra, 2018[66]	PTLD	P & A	8/8 matched unrelated donor vs. matched sib donor	SHR=1.51, p=0.19	
Landgren, 2009[69]	PTLD	P & A	2+ HLA antigen–mismatched related or unrelated donor, no ATG, no selective T-cell depletion vs. matched sibling or 1 HLA-Ag mismatched relative	-	RR=0.9 (0.3-2.2)
Landgren, 2009[69]	PTLD	P & A	2+ HLA antigen–mismatched related or unrelated donor, ATG and/or selective T-cell depletion vs. matched sibling or 1 HLA-Ag mismatched relative	-	**RR=3.8 (2.4-6.1)**
Fujimoto, 2019[54]	PTLD	P & A	MMRD vs. MRD	**HR=10.4 (6.35-17.1); p<0.001**	**HR=4.39 (2.39-8.07); p<0.001**
			MURD vs. MRD	**HR=4.89 (3.07-7.79); p<0.001**	**HR=4.08 (2.39-6.99); p<0.001**
			MMURD vs. MRD	**HR=5.46 (2.88-10.3); p<0.001**	**HR=3.20 (1.58-6.47); p=0.001**
			CB vs. MRD	**HR=7.24 (4.56-11.5); p<0.001**	**HR=8.03 (4.72-13.7); p<0.001**
Cohen, 2005[45]	PTLD	P	HLA mismatched vs. matched	OR=1.49 (0.39-5.59)	
Gao, 2019[55]	PTLD	P & A	Haploidentical donors vs. Matched sibling donors	**p<0.001**	HR=2.0 (0.5-8.3); p=0.350
Uhlin, 2014[93]	PTLD	P & A	HLA mismatched vs. matched	NR	**SHR=5.89 (2.43-14.3); p<0.001**
Liu, 2013[26]	PTLD	P & A	HLA matched vs. mismatched	**p=0.008**	p=0.691
Xuan, 2013[16]	PTLD	P & A	HLA matched vs. mismatched	p<0.001	NS
Brunstein, 2006[37]	EBV/PTLD	P & A	HLA, engrafted in doubles (5 of 6 vs. 6 of 6)		HR=0.2 (0.1-1.5) p=0.12
Brunstein, 2006[37]	EBV/PTLD	P & A	HLA, engrafted in doubles (3-4 of 6 vs. 6 of 6)	-	HR=0.9 (0.2-4.7) p=0.94
Sanz, 2014[87]	PTLD	P & A	HLA compatibility (6 of 6, 5 of 6, 4 of 6)	NR	NR (NS)
Kalra, 2018[66]	PTLD	P & A	8/8 matched vs ≤7/8 matched	SHR=1.30, p=0.34	
Xu, 2015[97]	PTLD	P & A	HLA disparity 2-3 loci vs.1 locus	HR=0.65 (0.18-2.34) p=0.509	
Van der Velden, 2013[94]	PTLD	A	HLA mismatched (Categories unspecified)	-	NR (NS)
Althubaiti, 2019[31]	PTLD	P	Degree of HLA match 10/10 vs. Others	p=0.497	-
sStyczynski, 2013[89]	PTLD	P & A	MMFD/haplo vs. MFD	HR=2.47 (1.17-5.17) p=0.015	-
Styczynski, 2013[89]	PTLD	P & A	MUD vs. MFD	HR=3.43 (2.07-5.74) p<0.001	-
Styczynski, 2013[89]	PTLD	P & A	MUD vs. MMUD	HR=9.72 (5.53-17.17) p<0.001	-
Styczynski, 2013[89]	PTLD	P & A	MMFD or unrelated donor vs. MFD	HR=4.11 (2.55-6.69) p<0.001	-
			**Graft source**		
Hiwarkar, 2013[58]	EBV	P	PBSC vs. Others	OR=1.8; p=NS	

Table S4: Risk factors for post-transplant EBV infection and for PTLD explored in the 77 retained studies

First author, year	Outcome	Study population	Risk factors explored	Estimate (95% CI); p-value	
				Univariate results	Multivariate results
Juvonen, 2007[65]	EBV	A	PBSC vs. BM	p=0.69	HR=1.42 (0.43-4.68); P=0.57
Patriarca, 2013[4]	EBV	A	BM vs. PBSC	p=0.511	-
Peric, 2011[84]	EBV	A	BM vs. PBSC	p=0.41	-
Garcia-Cadenas, 2015[56]	EBV	A	CB vs. others	p=0.46	-
Van Esser, 2001[95]	EBV	P & A	BM vs. PBSC	NS	-
Bogunia-Kubik, 2007[34]	EBV	P & A	(BM, PBSC)	NR	NR (NS)
Wang, 2019[96]	EBV	P &A	PB + BM vs. PB	**p=0.001**	**HR=7.89; p=0.003**
			BM vs. PB		**HR=18.69; p<0.001**
Tsoumakas, 2019[92]	EBV	P	PBSC vs. BM	**HR=2.55 (1.05-6.15); p=0.038**	**HR=2.51 (1.04-6.05); p=0.041**
Cesaro, 2010[41]	EBV	P	BM vs. CB	**p=0.047**	
Chiereghin, 2016[42]	EBV	P	BM vs. PBSC vs. CB	p=0.529	-
Chiereghin, 2019[43]	EBV	P & A	PBSC vs. CB vs. BM	p= 0.597	-
Marinho-Dias, 2019[75]	EBV	P & A	PBSC vs. CB or BM	OR=2.00 (0.37-11.1); p=0.414	-
Auger, 2014[33]	EBV	A	PBSC vs. UCB vs. BM	p=0.06	-
Islam, 2010[61]	EBV	P & A	BM vs. PBSC vs. UCB (Non-malignant group, Malignant group)	(p=1, p=0.69)	-
Garcia-Cadenas, 2015[56]	PTLD	A	CB vs. others	p=0.88	-
Kalra, 2018[66]	PTLD	P & A	BM vs. PBSC	Could not be analyzed	
Kalra, 2018[66]	PTLD	P & A	CB vs. PBSC	SHR=2.20, p=0.12	
Uhlin, 2014[93]	PTLD	P & A	(BM, PBSC, CB)	NR	NR (NS)
Styczynski, 2013[89]	PTLD	P & A	CB vs. Others	HR=3.61 (1.74-7.46) p<0.001	-
Ali, 2019[30]	PTLD	P	PBSC vs. CB vs. BM	**p= 0.017**	-
			**Graft content**		
Christopeit, 2013[44]	EBV	A	$CD3^+$ graft content ≥ vs. < median		**OR=0.111 (0.02-0.78); p=0.027**
Zhou, 2020[100]	EBV	P & A	$CD3^+$ cell counts, $x10^8$/kg (>1.92 vs. ≤1.9)	HR=0.608(0.246-1.500); p=0.280	NI
Van Esser, 2001[95]	EBV	P & A	Number of $CD3^+$ infused	NS	-
Christopeit, 2013[44]	EBV	A	$CD3^+CD8^+$ graft content ≥ vs. < median		**OR=0.05 (0.01-0.43); p=0.007**
Christopeit, 2013[44]	EBV	A	$CD3^+CD4^+$ graft content ≥ vs. < median		OR=0.48 (0.09-2.63); p=0.395
Zhou, 2020[100]	EBV	P & A	$CD4^+$ cell counts, $\times10^8$/kg (>1.12 vs. ≤1.12)	HR=0.608 (0.246-1.500); p=0.280	NI
Zhou, 2020[100]	EBV	P & A	$CD8^+$ cell counts, $\times10^8$/kg (>0.83 vs. ≤0.83)	HR=0.432 (0.173-1.081); p=0.073	HR=0.731 (0.190-2.667); p=0.615
Zhou, 2020[100]	EBV	P & A	$CD4^+/CD8^+$ ratio, $\times10^8$/kg (>1.38 vs. ≤1.38)	HR=1.752 (0.710-4.323); p=0.224	NI
Christopeit, 2013[44]	EBV	A	$CD16^+$ graft content ≥ vs. < median		OR=0.31 (0.05-1.85); p=0.200
Christopeit, 2013[44]	EBV	A	$CD19^+$ graft content ≥ vs. < median		OR=0.48 (0.09-2.63); p=0.395
Christopeit, 2013[44]	EBV	A	$CD34^+$ graft content ≥ vs. < median		OR=1.8 (0.40-8.18); p=0.447
Peric, 2011[84]	EBV	A	$CD34^+$ cell count ($x10^6$/kg recipient body weight)	p=0.52	-
Van Esser, 2001[95]	EBV	P & A	$CD34^+$ cell count of the graft (>1,35$x10^6$/kg)	**HR=2.4 (1.4-4.1); P=0.001**	**HR=2.6 (1.5-4.6); p=0.001**
Dumas, 2013[49]	EBV	P & A	Number of $CD34^+$ cells infused	p>0.15	-
Sanz, 2014[87]	EBV	P & A	Number of $CD34^+$ cells infused	NR	NR (NS)
Zhou, 2020[100]	EBV	P & A	$CD34^+$ cell counts, $\times10^8$/kg (>3.85 vs. ≤3.85)	HR=1.000 (0.411-2.431); p>.99	NI
Dumas, 2013[49]	EBV	P & A	Number of nucleated cells infused	p>0.15	-
Laberko, 2017[68]	EBV	P & A	Dose of α/β T cells > vs. < Median	p=0.70	-
Laberko, 2017[68]	EBV	P & A	B cell dose > vs. < median	p=0.30	-
Van Esser, 2001[95]	EBV	P & A	Number of MNCs infused	NS	-
Zhou, 2020[100]	EBV	P & A	MNCs from BM, $\times10^8$/kg (>5.60 vs. ≤5.60)	HR=1.393 (0.571-3.394); p=0.466	NI
Zhou, 2020[100]	EBV	P & A	MNCs from PBSCs, $\times10^8$/kg (>5.03 vs. ≤5.03)	HR=0.882 (0.363-2.146); p=0.783	NI
Van Esser, 2001[95]	EBV	P & A	Number of CFU-GMs infused	NS	-
Xuan, 2012[98]	EBV	P & A	Donor lymphocyte infusion (Yes vs. No)	NS	NS
Carpenter, 2010[22]	EBV	P & A	Graft content of $CD3^-$ (Categories unspecified)	NR	NR (NS)
Carpenter, 2010[22]	EBV	P & A	Graft content of $CD34^-$ (Categories unspecified)	NR	NR (NS)
Sanz, 2014[87]	EBV	P & A	Number of TNC cells infused	NR	NR (NS)
Cesaro, 2010[41]	EBV	P	Median TNC infused (bone marrow) ≥ vs. <$4x10^8$/kg	**p=0.02**	
Cesaro, 2010[41]	EBV	P	Dose of TNC infused (bone marrow) ≥ vs. <median	**p=0.03**	

Table S4: Risk factors for post-transplant EBV infection and for PTLD explored in the 77 retained studies

First author, year	Outcome	Study population	Risk factors explored	Estimate (95% CI); p-value	
				Univariate results	Multivariate results
Peric, 2012[83]	EBV	A	Median $CD34^+$ cell count ($x10^5$/kg recipient body weight)	p=0.94	
Peric, 2012[83]	EBV	A	Median total nucleated cells infused ($x10^7$/kg recipient body weight)	p=0.18	
Islam, 2010[61]	EBV	P & A	CD34 count < 2 $x10^6$/kg vs. ≥ 2 $x10^6$/kg (Non-malignant group, Malignant group)	(p=0.17, p=0.45)	-
Comoli, 2007[46]	EBV	P & A	Number of $CD34^+$ cells present in the graft	NS (NR)	-
Islam, 2010[61]	EBV	P & A	D100 Lymph count <1000/mm^3 vs. ≥ 1000/mm^3 (Non-malignant group, Malignant group)	(p=0.053, p=1)	-
Sanz, 2014[87]	EBV	P & A	Number of TNC cells infused	NR	NR (NS)
Uhlin, 2014[93]	PTLD	P & A	Nucleated dose (10^8/kg)	NR	NR (NS)
Buyck, 2009[39]	PTLD	P & A	Total nucleated cell count per $1x10^8$/kg	HR=1.01 (0.90-1.14); p=0.82	-
Xu, 2015[97]	PTLD	P & A	TLC for infusion	HR=0.89 (0.18-4.27) p=0.879	
Xu, 2015[97]	PTLD	P & A	$CD3^+$ cells count for infusion ≥ vs. < median	HR=0.65 (0.13-3.42) p=0.614	
Xu, 2015[97]	PTLD	P & A	$CD4^+$ cells count for infusion ≥ vs. < median	HR=0.75 (0.22-2.62) p=0.657	
Xu, 2015[97]	PTLD	P & A	$CD34^+$ cells count for infusion ≥ vs. < median	HR=0.79 (0.32-1.95) p=0.610	
Sanz, 2014[87]	PTLD	P & A	Number of $CD34^+$ cells infused	NR	NR (NS)
Uhlin, 2014[93]	PTLD	P & A	Number of $CD34^+$ cells infused	NR	NR (NS)
			**Conditioning regimen & GvHD prophylaxis/treatment**		
Garcia-Cadenas, 2015[56]	EBV	A	MAC vs. RIC	p=0.49	-
Patriarca, 2013[4]	EBV	A	RIC vs. MAC	p=0.186	-
Burns, 2016[38]	EBV	P & A	MAC vs. RIC	HR=0.76 (0.44-1.29); p=0.309	-
Christopeit, 2013[44]	EBV	A	RIC/NMAC vs. MAC		OR=0.257 (0.042-1.573); p=0.142
Cohen, 2005[45]	EBV	P	RIC vs. CIC	**OR=5.66 (2.00-15.99)**	NR
Hiwarkar, 2013[58]	EBV	P	RIC vs. others	**OR=2.1; p<0.05**	NS
Sanz, 2014[87]	EBV	P & A	RIC vs. MAC	NR	**SHR=6.0 (2.0-17.6); p=0.001**
Marinho-Dias, 2019[75]	EBV	P & A	MAC vs. RIC	NR (at day +150 post-transplantation)	NS (NR)
Ru, 2020[85]	EBV	P & A	RIC vs. MAC	HR=1.049 (0.782–1.406); p=0.750	NI
Liu, 2013[73]	EBV	P & A	Intensified MAC vs. Standard MAC	**p=0.006**	**HR=1.72 (1.03-2.88); P=0.038**
Lin, 2019[71]	EBV	P & A	Intensified conditioning vs. Standard MAC		**HR=1.73 (1.18-2.54); p=0.005**
Dumas, 2013[49]	EBV	P & A	RIC with ATG vs. MAC	**p=0.03**	NS
Dumas, 2013[49]	EBV	P & A	RIC without ATG vs. MAC	p=0.26	NS
Bogunia-Kubik, 2007[34]	EBV	P & A	(MAC, RIC)	NR	NR (NS)
Jaskula, 2010[64]	EBV	P & A	(MAC, RIC)	NR	NR (NS)
Auger, 2014[33]	EBV	A	MAC vs. RIC	NS	-
Chiereghin, 2016[42]	EBV	P	MAC vs. RIC	**p=0.013**	-
Chiereghin, 2019[43]	EBV	P & A	MAC vs. RIC	**p=0.023**	**-**
Meijer, 2004[76]	EBV	A	MAC vs. NMAC	**p<0.05**	**-**
Buyck, 2009[39]	PTLD	P & A	RIC vs. No RIC	**HR=8.8 (1.47-52.7); p=0.02**	HR=5.00 (0.75-33.30); p=0.1
Garcia-Cadenas, 2015[56]	PTLD	A	MAC vs. RIC	p=0.97	-
Fujimoto, 2019[54]	PTLD	P & A	RIC vs. MAC	**HR=2.00 (1.56-2.55); p<0.001**	HR=0.82 (0.60-1.12); p=0.22
Xuan, 2013[16]	PTLD	P & A	Standard vs. intensified conditioning	**p=0.003**	**HR=4.46 (1.20-16.61) p=0.026**
Brunstein, 2006[37]	EBV/PTLD	P & A	NMAC without ATG vs. MAC	-	HR=0.7 (0.1-6.5) p=0.51
Brunstein, 2006[37]	EBV/PTLD	P & A	NMAC with ATG vs. MAC	-	**HR=15.4 (2.0-116.1) p<0.01**
Liu, 2013[26]	PTLD	P & A	Intensified MAC vs. Standard MAC	**p=0.016**	**p=0.018**
Sanz, 2014[87]	PTLD	P & A	RIC vs. MAC	**p=0.001**	**SHR=5.5 (1.8-17.1); p=0.003**
Uhlin, 2014[93]	PTLD	P & A	RIC vs. No RIC	NR	**SHR=3.25 (1.53-6.89); p=0.002**
Van der Velden, 2013[94]	PTLD	A	MAC without ATG	-	**OR=2.6 (1.05-7.15) p=0.01**
Van der Velden, 2013[94]	PTLD	A	NMAC with ATG	-	OR=2.1 (0.92-4.8) p=0.08
Althubaiti, 2019[31]	PTLD	P	MAC vs. non-MAC	p=0.052	-
Kullberg-Lindh, 2011[67]	EBV	P	Use of TBI (Yes vs. No)	**slope=0.98; p=0.05**	**slope=1.60; p=0.001**
Ru, 2020[85]	EBV	P & A	TBI (Yes vs. No)	HR=1.037 (0.796–1.352); p=0.786	NI
Garcia-Cadenas, 2015[56]	EBV	A	Use of TBI (Yes vs. No)	**p=0.00**	NS

Table S4: Risk factors for post-transplant EBV infection and for PTLD explored in the 77 retained studies

First author, year	Outcome	Study population	Risk factors explored	Estimate (95% CI); p-value	
				Univariate results	Multivariate results
Christopeit, 2013[44]	EBV	A	Use of TBI (Yes vs. No)		OR=0.556 (0.11-2.9); p=0.486
Juvonen, 2007[65]	EBV	A	Use of TBI (Yes vs. No)	p=1.00	HR=0.79 (0.36-1.75); P=0.57
Zhou, 2020[100]	EBV	P & A	Bu/Cy vs. Bu/Flu	HR=0.832 (0.337-2.057); p=0.690	NI
Burns, 2016[38]	EBV	P & A	Cy TBI vs. Flu Mel	HR=0.63 (0.37-1.08); p=0.092	HR=0.69 (0.35-1.36); P=0.284
Burns, 2016[38]	EBV	P & A	BEAM+/ - Flu vs. Flu Mel	No events	No events
Burns, 2016[38]	EBV	P & A	Other vs. Flu Mel	HR=0.81 (0.25-2.56); p=0.714	HR=0.27 (0.05-1.36); P=0.112
Cesaro, 2010[41]	EBV	P	Use of TBI (Yes vs. No)	p=0.1	
Comoli, 2007[46]	EBV	P & A	Use of TBI (Yes vs. No)	NS (NR)	-
Hoshino, 2001[60]	EBV	P & A	Use of TBI (Yes vs. No)	p=0.5592	-
Peric, 2012[83]	EBV	A	Use of TBI (Yes vs. No)	p=1.00	
Liu, 2013[73]	EBV	P & A	Use of Flu (Yes vs. No)	NS	NS
Dumas, 2013[49]	EBV	P & A	Use of Flu or Bu	p>0.15	-
Auger, 2014[33]	EBV	A	Use of Flu (Yes vs. No)	NS	-
Garcia-Cadenas, 2015[56]	PTLD	A	Use of TBI (Yes vs. No)	p=0.1	NS
Uhlin, 2014[93]	PTLD	P & A	Use of TBI (Yes vs. No)	NR	NR (NS)
Uhlin, 2014[93]	PTLD	P & A	Use of Bu (Yes vs. No)	NR	NR (NS)
Xuan, 2013[16]	PTLD	P & A	Use of Flu (Yes vs. No)	NS	NS
Hoegh-Petersen, 2011[59]	PTLD	A	Flu+Bu+ATG	p=0.97	
Hoegh-Petersen, 2011[59]	PTLD	A	Flu+Bu+TBI+ATG		
Hoegh-Petersen, 2011[59]	PTLD	A	Cy+ATG		
Hoegh-Petersen, 2011[59]	PTLD	A	Flu+Mel+ATG		
Hoegh-Petersen, 2011[59]	PTLD	A	VP16+TBI+ATG		
Zhou, 2020[100]	EBV	P & A	Type of ATG for GVHD prophylaxis (ATG-T vs. ATG-F)	**HR=4.378 (1.360-14.093); p=0.013**	HR=2.981 (0.522-17.031); p=0.219
Figgins, 2019[53]	EBV	A	Use of ATG (Yes vs. No)	Cumulative incidence (20% vs. 9%); p=0.08	-
Marinho-Dias, 2019[75]	EBV	P & A	Use of ATG (Yes vs. No)	OR=2.91 (0.70-12.1); p=0.135	-
Mountjoy, 2020[77]	EBV	A	Use of ATG (Yes vs. No)	Proportion (18.6% vs. 8.8%); p=0.08	-
Peric, 2012[83]	EBV	A	Use of ATG (Yes vs. No)	p=0.57	
Kullberg-Lindh, 2011[67]	EBV	P	Use of ATG (Yes vs. No)	**slope=1.20; p=0.01**	**slope=1.34; p=0.004**
Cesaro, 2004[40]	EBV	P	Use of ATG (Yes vs. No)	**p=0.0006**	**HR=13.0 (2-96); p=0.01**
Chiereghin, 2016[42]	EBV	P	*In vivo* TCD with ATG (Yes vs. No)	p=0.081	-
Juvonen, 2007[65]	EBV	A	Use of ATG (Yes vs. No)	**p<0.0001**	**HR=5.78 (2.47-13.5); p<0.001**
Fan, 2016[52]	EBV	P & A	Use of ATG (Yes vs. No)	NR	**OR=7.69 (1.17-50.49); p=0.034**
Liu, 2013[73]	EBV	P & A	Use of ATG (Yes vs. No)	**p<0.001**	**HR=14.08 (6.02-32.92); p<0.001**
Laberko, 2017[68]	EBV	P & A	Horse ATG (Yes vs. No)	-	HR= 2.47 (0.95-6.38); p=0.063
Laberko, 2017[68]	EBV	P & A	Rabbit ATG (Yes vs. No)	-	HR= 1.22 (0.467-3.18); p= 0.69
Christopeit, 2013[44]	EBV	A	Use of ATG (Yes vs. No)		OR=0.83 (0.17-4.01); p=0.820
Peric, 2011[84]	EBV	A	Use of ATG (Yes vs. No)	**p=0.006**	**SHR=4.9 (1.1-21.0); p=0.03**
Gao, 2019[55]	EBV	P & A	Use of ATG (Yes vs. No)	**p<0.001**	**HR=6.3 (1.6-24.0); p=0.008**
Düver, 2020[50]	EBV	P	Use of ATG (Yes vs. No)	**p<0.001**	**OR=10.68 (1.15–98.86); p=0.037**
Ru, 2020[85]	EBV	P & A	Use of ATG (Yes vs. No)	**HR=5.125 (3.247–8.089); p<0.001**	**HR=4.288(2.638-6.97); p<0.001**
Patriarca, 2013[4]	EBV	A	No ATG vs. Low dose vs. Standard dose	**p=0.002**	NS
Cohen, 2005[45]	EBV	P	ATG vs. Campath	**OR=2.72 (1.10-6.73)**	OR=2.09 (0.83-5.29)
Elmahdi, 2016[51]	EBV	P	Dose of ATG (15 mg/kg vs. 10 mg/kg)	HR=1.61 (0.62-3.97); p=0.331	
Elmahdi, 2016[51]	EBV	P	ATG median (4 wk) ≥ vs. < 13,7 µg/mL	HR=0.60 (0.28-1.57); p=0.249	
Elmahdi, 2016[51]	EBV	P	ATG threshold (4 wk) ≥ vs. < 6,2 µg/mL	HR=0.56 (0.21-1.48); p=0.245	
Neumann, 2018[78]	EBV	A	Campath vs. ATG	p=0.317	Campath group and ATG group have been matched according to the variables age, diagnosis, and conditioning regimen
Hoshino, 2001[60]	EBV	P & A	Use of ATG (Yes vs. No)	**p=0.0005**	-
Islam, 2010[61]	EBV	P & A	Previous therapy: Non ATG vs. ATG (Non-malignant group)	**OR=0.286; p=0.04**	-

Table S4: Risk factors for post-transplant EBV infection and for PTLD explored in the 77 retained studies

First author, year	Outcome	Study population	Risk factors explored	Estimate (95% CI); p-value	
				**Univariate results**	**Multivariate results**
Auger, 2014[33]	EBV	A	Use of ATG (Yes vs. No)	**p=0.004**	-
D'Aveni, 2011[48]	EBV	P & A	Use of ATG (Yes vs. No)	**p=0.005**	-
Zhou, 2020[101]	PTLD	P & A	Type of ATG for GVHD prophylaxis (ATG-T vs. ATG-F)	Cumulative incidence 28.4% (19.0–38.5%) vs. 25.8% (12.6–41.0%); p=0.717	-
Ali, 2019[30]	PTLD	P	Use of ATG (Yes vs. No)	p=0.055	-
Landgren, 2009[69]	PTLD	P & A	Use of ATG (Yes vs. No)	-	**RR=3.8 (2.5-5.8)**
Van der Velden, 2013[94]	PTLD	A	Use of ATG (Yes vs. No)	-	**OR=2.4 (1.3-4.2) p=0.001**
Liu, 2013[26]	PTLD	P & A	Use of ATG (Yes vs. No)	**p=0.001**	**p=0.038**
Xuan, 2013[16]	PTLD	P & A	Use of ATG (Yes vs. No)	p<0.001	**HR=13.03 (1.67-101.58); p=0.014**
Fujimoto, 2019[54]	PTLD	P & A	Use of ATG in conditioning regimen (Yes vs. No)	**HR=7.76 (6.03-9.99); p<0.001**	**HR=6.13 (4.33-8.68); p<0.001**
Fujimoto, 2019[54]	PTLD	P & A	Use of ATG for GvHD treatment (Yes vs. No) ǂ	**HR=6.87 (4.00-11.8); p<0.001**	**HR=2.09 (1.17-3.72); p=0.01**
Gao, 2019[55]	PTLD	P & A	Use of ATG (Yes vs. No)	**p=0.001**	HR=2.9 (0.3-27.5); p=0.350
Lin, 2019[71]	EBV	P & A	ATG dose (10.0 mg/kg vs. 7.5 mg/kg)		**HR=2.02 (1.37-2.97); p<0.001**
Issa, 2019[62]	EBV	A	r-ATG (6 mg/kg vs. 4.5 mg/kg)	**Cumulative incidence (0.18 [0.13-0.23] vs. 0.09 [0.05-0.15]; p=0.03)**	-
Cohen, 2005[45]	PTLD	P	Campath vs. ATG	OR=0.56 (0.15-2.05)	
Buyck, 2009[39]	PTLD	P & A	Number of prior courses of ATG (per course)	HR=10.39 (2.03-53.18); p=0.005	**HR=7.23 (1.67-31.32); p=0.008**
Buyck, 2009[39]	PTLD	P & A	Campath vs. ATG	HR=1.06 (0.12-9.46); p=0.96	-
Chiereghin, 2019[43]	EBV	P & A	In vivo T-cell depletion with ATG/ALG (Yes vs. No)	p=1.000	-
Althubaiti, 2019[31]	PTLD	P	In-vivo T-cell depletion (ATG or alemtuzumab)	p=0.120	-
Cesaro, 2004[40]	EBV	P	CsA vs. CsA+other	**p=0.004**	NS
Fan, 2016[52]	EBV	P & A	Mycophenolate mofetil + cyclosporine + prednisone vs. Mycophenolate mofetil + cyclosporine	NR	**OR=23.68 (1.92-291.45); p=0.013**
Peric, 2011[84]	EBV	A	CsA alone vs. CsA+MMF vs. CsA+MTX	p=0.85	-
Althubaiti, 2019[31]	PTLD	P	CsA/MTX vs. CsA with other combinations vs. MMF	p=0.261	-
Sanz, 2014[87]	EBV	P & A	(CsA + MMF, CsA + prednisone)	NR	NR (NS)
Islam, 2010[61]	EBV	P & A	CSA vs. CSA+MTX vs. Other (Non-malignant group, Malignant group)	(p=0.35, p=0.53)	-
Sanz, 2014[87]	PTLD	P & A	(CsA + MMF, CsA + prednisone)	NR	NR (NS)
Uhlin, 2014[93]	PTLD	P & A	CsA+MTX vs. Other	NR	NR (NS)
Garcia-Cadenas, 2015[56]	PTLD	A	GvHD prophylaxis (Yes vs. No)	p=0.16	-
Christopeit, 2013[44]	EBV	A	Mean (days 0-30) CsA (≥ vs. <201 ng/mL)		OR=3.00 (0.61-14.86); p=0.178
Christopeit, 2013[44]	EBV	A	CsA AUC (≥ vs. <6000 ng/mL x days)		**OR=6.07 (1.11-33.24); p=0.038**
Christopeit, 2013[44]	EBV	A	CsA/MTX vs. CsA/MPA		OR=3.67 (0.35-38.03); p=0.276
Hoshino, 2001[60]	EBV	P & A	Use of tacrolimus vs. CsA	p=0.643	-
Fujimoto, 2019[54]	PTLD	P & A	Use of tacrolimus vs. CsA	**HR=2.07 (1.59-2.69); p<0.001**	HR=0.82 (0.59-1.12); p=0.21
Xuan, 2012[98]	EBV	P & A	Early CsA withdrawal (Yes vs. No)	NS	NS
Comoli, 2007[46]	EBV	P & A	GvHD prophylaxis (Categories unspecified)	NS (NR)	-
Laberko, 2017[68]	EBV	P & A	Post-HSCT GvHD prophylaxis (Yes vs. No)	p=0.45	-
Carpenter, 2010[22]	EBV	P & A	Alemtuzumab (In vitro vs. In vivo)	NR	HR=1.63 (0.83-3.21); p=0.160
Rustia, 2016[86]	EBV	P	Alemtuzumab (Yes vs. No)	p=0.172	-
Althubaiti, 2019[31]	PTLD	P	Use of alemtuzumab (Yes vs. No)	p=0.500	-
Gao, 2019[55]	EBV	P & A	Use of fludarabine (Yes vs. No)	p=0.713	NI
Gao, 2019[55]	PTLD	P & A	Use of fludarabine (Yes vs. No)	**p=0.022**	**HR=3.8 (1.4-10.6); p=0.010**
Comoli, 2007[46]	EBV	P & A	Post-transplant steroid	NS (NR)	-
Liu, 2013[26]	EBV	P & A	Use of steroid therapy (Yes vs. No)	**p=0.004**	p=0.733
Elmahdi, 2016[51]	EBV	P	Steroid (Yes vs. No)	HR=3.66 (1.05-12.75); p=0.065	
Juvonen, 2007[65]	EBV	A	High dose steroid (≥ vs. <2 mg/kg/day)ǂ	**p<0.0001**	HR=0.73 (0.25-2.08); P=0.55
Liu, 2013[26]	PTLD	P & A	Use of steroid therapy (Yes vs. No)	0.976	0.433

Table S4: Risk factors for post-transplant EBV infection and for PTLD explored in the 77 retained studies

First author, year	Outcome	Study population	Risk factors explored	Estimate (95% CI); p-value	
				Univariate results	Multivariate results
Islam, 2010[61]	EBV	P & A	Previous therapy: 0-2 chemo courses vs. ≥ 3 chemo courses (Malignant group)	p=1	
			**T-cell depletion**		
Bordon, 2012[36]	EBV	P	*In vivo* TCD (Yes vs. No)	**p=0.02**	**p=0.04**
Torre-Cisneros, 2004[90]	EBV	P & A	Use of $CD4^+$ lymphocyte-depleted graft	**HR=11.5 (5.8-22.8); p<0.0001**	**HR=11.5; (5.8-22.8); p<0.0001**
Van Esser, 2001[95]	EBV	P & A	TCD without ATG vs. Non-TCD	**HR=1.5 (0.8-2.7); p=0.02**	HR=1.5 (0.8-2.9); p=0.3
Van Esser, 2001[95]	EBV	P & A	TCD with ATG vs. Non-TCD	**HR=3.5 (1.8-6.9); p<0.001**	**HR=3.4 (1.6-7.1); p=0.001**
Cohen, 2005[45]	EBV	P	*In vitro* TCD (Yes vs. No)	**OR=0.17 (0.04-0.78)**	OR=0.40 (0.08-2.01)
Düver, 2020[50]	EBV	P	*In vitro* T-cell depletion (Yes vs. No)	p=0.37	NI
Hiwarkar, 2013[58]	EBV	P	*In vivo* TCD (Yes vs. No)	**OR=2.6; p<0.05**	NS
Zallio, 2013[23]	EBV	A	*In vivo* TCD (Yes vs. No)	**p=0.002**	NS
Sirvent-von Bueltzingsloewen, 2002[88]	EBV	P & A	Lymphocyte depletion (Yes vs. No)	**p<0.01**	NS
Cohen, 2005[45]	EBV	P	Use of serotherapy (Yes vs. No)	OR=2.27 (0.49-10.58)	-
Comoli, 2007[46]	EBV	P & A	Use of serotherapy (Categories unspecified)	NS (NR)	-
Garcia-Cadenas, 2015[56]	EBV	A	T-cell depletion in the 6 months before transplant (Yes vs. No)	**p<0.01**	NS
Garcia-Cadenas, 2015[56]	PTLD	A	T-cell depletion 6 months prior SCT (Yes vs. No)	p=0.16	-
			**Method of T-cell depletion**		
Landgren, 2009[69]	PTLD	P & A	Broad lymphocyte depletion vs. No T-cell depletion	-	**RR=3.1 (1.2-6.7)**
Landgren, 2009[69]	PTLD	P & A	Selective T-cell depletion vs. No T-cell depletion	-	**RR=9.4 (6.0-14.7)**
Landgren, 2009[69]	PTLD	P & A	Broad lymphocyte depletion		
Landgren, 2009[69]	PTLD	P & A	Alemtuzumab MoAb vs. No T-cell depletion	-	RR=3.1 (0.7-8.4)
Landgren, 2009[69]	PTLD	P & A	Elutriation/density gradient centrifugation vs. No T-cell depletion	-	RR=3.2 (0.8-8.8)
Landgren, 2009[69]	PTLD	P & A	Selective T-cell depletion		
Landgren, 2009[69]	PTLD	P & A	Anti-T or anti-T + NK MoAb vs. No T-cell depletion	-	**RR=8.4 (5.1-13)**
Landgren, 2009[69]	PTLD	P & A	SRBC rosetting vs. No T-cell depletion	-	**RR=14.6 (5.9-31)**
Landgren, 2009[69]	PTLD	P & A	Lectins with/without SRBC or anti-T MoAb vs. No T-cell depletion	-	**RR=15.8 (7.2-32)**
Landgren, 2009[69]	PTLD	P & A	Unclassified/unknown method vs. No T-cell depletion	-	RR=6.0 (0.96-20)
Van der Velden, 2013[94]	PTLD	A	(CD3/CD19 depletion, CD34 selection)	-	NR (NS)
			**Graft-versus-host disease**		
Cesaro, 2004[40]	EBV	P	aGvHD Grade 0-I vs. II-IV	p=1.0	-
Juvonen, 2007[65]	EBV	A	aGvHD Grade ≥ IIII‡	**p<0.0001**	**HR=1.70 (1.11-2.62); P=0.015**
Torre-Cisneros, 2004[90]	EBV	P & A	aGvHD Grade ≥ IIII	HR=1.1 (0.6-2); p=0.78	NI
Düver, 2020[50]	EBV	P	aGvHD (Grade III-IV vs. None or Grade ≤II)	**p=0.021**	NS (NR)
Zhou, 2020[100]	EBV	P & A	aGVHD Grade III-IV (Yes vs. No)	HR= 2.565 (0.678-9.699); p=0.165	NI
Zhou, 2020[100]	EBV	P & A	aGVHD Grade I-II (Yes vs. No)	HR=1.057 (0.427-2.621); p=0.904	NI
Zhou, 2020[100]	EBV	P & A	aGVHD (Grade III-IV vs. Grade I-II)	HR=2.235 (0.571-8.754); p=0.248	NI
Ru, 2020[85]	EBV	P & A	aGvHD (Grade II-IV vs. None or Grade I)	HR= 1.336 (0.968-1.845); p= 078	HR=1.257 (0.891-1.775); p= 0.193
Burns, 2016[38]	EBV	P & A	aGvHD Grade ≥ II	HR=1.53 (0.91-2.57); p=0.112	-
Sirvent-von Bueltzingsloewen, 2002[88]	EBV	P & A	aGvHD Grade ≥II	**p<0.01**	**OR=3.4 (1.2-9.7)**
Hiwarkar, 2013[58]	EBV	P	aGvHD Grade≥ II	**OR=3.6; p<0.001**	**Significant but NR**
Garcia-Cadenas, 2015[56]	EBV	A	aGvHD Grade ≥ II‡	p=0.48	-
Patriarca, 2013[4]	EBV	A	aGvHD Grade ≥II	p=0.082	NS
Liu, 2013[73]	EBV	P & A	aGvHD Grade ≥II	NS	NS
Peric, 2011[84]	EBV	A	aGvHD Grade 0-I vs. II vs. III-IV	p=0.36	-
Gao, 2019[55]	EBV	P & A	aGvHD (Yes vs. No)	**p=0.001**	HR=1.0 (0.7-1.6); p=0.960
Marinho-Dias, 2019[75]	EBV	P & A	aGvHD (Yes vs. No)	OR=3.09 (0.75-12.8); p=0.170	-
Zhou, 2020[100]	EBV	P & A	aGVHD (Yes vs. No)	HR=1.791 (0.631-5.080); p=0.273	NI
Elmahdi, 2016[51]	EBV	P	aGvHD (Yes vs. No)	**HR=3.29 (1.26-8.58); p=0.015**	**HR=3.29 (1.26-8.58); p=0.015**
Cohen, 2005[45]	EBV	P	aGvHD (Yes vs. No)	**OR=2.53 (1.07-5.97)**	**OR=2.20 (2.12-15.08)**
Kullberg-Lindh, 2011[67]	EBV	P	aGvHD (Yes vs. No)	Slope=0.71; p=0.24	Slope=0.48; p=0.34
Omar, 2009[80]	EBV	P & A	aGvHD (Yes vs. No)	NR	**p=0.009**
Jaskula, 2010[64]	EBV	P & A	aGvHD (Categories unspecified)	NR	NR (NS)
Sanz, 2014[87]	EBV	P & A	aGVHD (Categories unspecified)	NR	NR (NS)

Table S4: Risk factors for post-transplant EBV infection and for PTLD explored in the 77 retained studies

First author, year	Outcome	Study population	Risk factors explored	Estimate (95% CI); p-value	
				Univariate results	Multivariate results
Chiereghin, 2016[42]	EBV	P	aGvHD (Absent vs. Grade I vs. Grade ≥II)	p=0.846	-
Chiereghin, 2019[43]	EBV	P & A	aGvHD (Absent vs. Grade I vs. Grade≥II)	p=0.986	-
Comoli, 2007[46]	EBV	P & A	aGvHD (Categories unspecified)	NS (NR)	-
Peric, 2012[83]	EBV	A	aGvHD Grade 0-II vs. Grade III-IV	p=0.69	
Islam, 2010[61]	EBV	P & A	aGvHD: None vs. Grade I vs. Grade II (Non-malignant group, Malignant group)	(p=0.44, p=0.70)	-
Cesaro, 2004[40]	EBV	P	cGvHD (Yes vs. No)	p=0.8	-
Cohen, 2005[45]	EBV	P	cGvHD (Yes vs. No)	OR=1.38 (0.34-5.63)	-
Kullberg-Lindh, 2011[67]	EBV	P	cGvHD (Yes vs. No)	Slope=-0.86; p=0.09	**Slope=-1.12; p=0.023**
Ru, 2020[85]	EBV	P & A	cGvHD (Yes vs. No)	**HR=1.436 (1.051-1.96); p=0.023**	**HR= 1.413 (1.013-1.971); p= 0.042**
Liu, 2013[73]	EBV	P & A	cGvHD (Yes vs. No)	NS	NS
Patriarca, 2013[4]	EBV	A	cGvHD (Mild to severe vs. Absent)	p=0.527	
Sanz, 2014[87]	EBV	P & A	cGVHD (Categories unspecified)	NR	NR (NS)
Chiereghin, 2016[42]	EBV	P	cGvHD (Absent vs. Mild to severe)	p=0.369	-
Chiereghin, 2019[43]	EBV	P & A	cGvHD (Absent vs. Mild to severe)	p=0.467	-
Islam, 2010[61]	EBV	P & A	cGvHD: None vs. Limited vs. Extensive (Non-malignant group, Malignant group)	(p=1, p=0.71)	-
Laberko, 2017[68]	EBV	P & A	GvHD (Yes vs. No)	**p=0.02**	**HR= 1.97 (1.04-3.72); p= 0.037**
Zallio, 2013[23]	EBV	A	GvHD (Yes vs. No)	**p=0.037**	NS
Fujimoto, 2019[54]	PTLD	P & A	aGvHD Grade II-IV (Yes vs. No) ǂ	**HR=1.83 (1.43-2.35); p<0.001**	**HR=1.93 (1.48-2.52); p<0.001**
Landgren, 2009[69]	PTLD	P & A	aGvHD Grade ≥ IIǂ	-	**RR=1.7 (1.2-2.5)**
Uhlin, 2014[93]	PTLD	P & A	aGvHD Grade ≥ II	NR	**SHR=2.65 (1.32-5.35); p=0.006**
Liu, 2013[26]	PTLD	P & A	aGvHD Grade ≥ II	p=0.998	0.836
Xuan, 2013[16]	PTLD	P & A	aGvHD Grade ≥ II	NS	NS
Xu, 2015[97]	PTLD	P & A	aGvHD Grade ≥ III	HR=1.31 (0.11-15.88); p=0.835	
Garcia-Cadenas, 2015[56]	PTLD	A	aGvHD Grade ≥ II	p=0.7	-
Van der Velden, 2013[94]	PTLD	A	aGvHD Grade ≥ II	-	NR (NS)
Gao, 2019[55]	PTLD	P & A	aGvHD (Yes vs. No)	p=0.134	HR=1.4 (0.5-3.8); p=0.480
Cohen, 2005[45]	PTLD	P	aGvHD (Yes vs. No)	OR=7.71 (95% CI:1.57-38.0)	
Sanz, 2014[87]	PTLD	P & A	aGVHD (Categories unspecified)	NR	NR (NS)
Kalra, 2018[66]	PTLD	P & A	aGvHD Grade II-IV or chronic NST (Yes vs. No)	**SHR=0.45; p=0.01**	**SHR=0.47, p=0.04**
Xuan, 2013[16]	PTLD	P & A	cGvHD (Yes vs. No)	NS	NS
Landgren, 2009[69]	PTLD	P & A	cGvHD (Moderate/severe or clinical extensive) ǂ	-	**RR=2.0 (1.1-3.2)**
Liu, 2013[26]	PTLD	P & A	cGvHD (None vs. Limited vs. Extensive)	0.319	0.842
Sanz, 2014[87]	PTLD	P & A	cGVHD (Categories unspecified)	NR	NR (NS)
			**Immunological reconstitution after HSCT**		
Auger, 2014[33]	EBV	A	Median of $CD34^+$ cells ($x10^6$/kg)	NS	-
Comoli, 2007[46]	EBV	P & A	$CD3^+$ T cells at 2 months post-HSCT	NS (NR)	-
Comoli, 2007[46]	EBV	P & A	$CD3^+CD8^+$ T cells at 2 months post-HSCT	NS (NR)	-
Comoli, 2007[46]	EBV	P & A	$CD3^+CD4^+$ T cells at 2 months post-HSCT	NS (NR)	-
Patriarca, 2013[4]	EBV	A	Peripheral blood lymphocyte/µl at +1 month after HSCT (≥100 vs. <100)	p=0.636	-
Patriarca, 2013[4]	EBV	A	Peripheral blood lymphocyte/µl at +3 months after HSCT (≥100 vs. <100)	p=1.00	-
Patriarca, 2013[4]	EBV	A	Peripheral blood CD4+ lymphocyte/µl at +1 month after HSCT (≥50 vs. <50)	**p=0.001**	**OR=0.1 (0.02-0.48); p=0.004**
Patriarca, 2013[4]	EBV	A	Peripheral blood CD4+ lymphocyte/µl at +3 month after HSCT (≥50 vs. <50)	p=0.530	-
Peric, 2011[84]	EBV	A	Neutrophil recovery ANC>$0.5x10^9$/l (Continuous)	p=0.32	-
Liu, 2018[74]	EBV	A	$CD4^-CD8^-$ count at day 30: Lower count (< median) vs. Higher	p>0.1	Procedures of donor priming, graft harvesting, conditioning, and GvHD prophylaxis were all the same. The possible influences of other factors on the recovery of T lymphocytes were minimized.
Liu, 2018[74]	EBV	A	Count $V\delta2^+$ T cells at day 60: Lower count (< median) vs. Higher	p=0.078	
Liu, 2020[72]	EBV	A	$CD3^+$ cells recovery at day 30 post-transplantation	-	HR=2.181 (0.390-12.187); p=0.374

Table S4: Risk factors for post-transplant EBV infection and for PTLD explored in the 77 retained studies

First author, year	Outcome	Study population	Risk factors explored	Estimate (95% CI); p-value		
				Univariate results	Multivariate results	
Liu, 2020[72]	EBV	A	CD4$^+$ cells recovery at day 30 post-transplantation	-	HR=0.717 (0.212-2.429); p=0.593	
Liu, 2020[72]	EBV	A	CD8$^+$ cells recovery at day 30 post-transplantation	-	HR=0.499 (0.207-1.201); p=0.121	
Liu, 2020[72]	EBV	A	CD8$^+$ αβT cells recovery at day 30 post-transplantation	-	HR=0.736 (0.034-15.986); p=0.845	
Liu, 2020[72]	EBV	A	γδT cells recovery at day 30 post-transplantation	-	HR=2.069 (0.389-11.013); p=0.394	
Liu, 2020[72]	EBV	A	Vδ1$^+$ cells recovery at day 30 post-transplantation	-	HR=0.640 (0.237-1.730); p=0.379	
Liu, 2020[72]	EBV	A	Vδ2$^+$ cells recovery at day 30 post-transplantation	-	**HR=0.347 (0.161-0.747); p=0.007**	
Park, 2020[82]	EBV	P & A	Normal T-cell reconstitution vs. Abnormal T-cell reconstitution[*]	**Proportion (5.1% vs. 20.0%; p=0.045)**	-	
Yu, 2019[99]	EBV	P & A	NKp30 in 1-month post-transplant (1M) (% of total NK cells)	**beta=-0.078 (-0.119; -0.037); p= 0.000**	**HR= 0.957 (0.918-0.998); p= 0.04**	
Yu, 2019[99]	EBV	P & A	NKp46 in 1M (% of total NK cells)	**beta=-0.233 (-0.033; -0.013); p= 0.000**	NI	
Yu, 2019[99]	EBV	P & A	NKG2D in 1M (% of total NK cells)	**beta=-1.768 (-3.068; -0.467); p= 0.008**	NI	
Yu, 2019[99]	EBV	P & A	NKG2A$^-$ CD57$^+$ KIR$^+$ % in 1M	**beta=-0.152 (-0.256; -0.048); p= 0.004**	NI	
Yu, 2019[99]	EBV	P & A	NKG2A$^-$ CD57$^+$ KIR$^+$ CD107$^+$ % in 1M	beta=0.077 (0.987-1.300); p= 0.419	NI	
Xu, 2015[97]	PTLD	P & A	TLCs at day 30 after HSCT ≥ vs. < median	HR=0.48 (0.22-1.05) p=0.066		
Xu, 2015[97]	PTLD	P & A	CD3$^+$ cells count at day 30 after HSCT ≥ vs. < median	HR=0.50 (0.13-1.96) p=0.322		
Xu, 2015[97]	PTLD	P & A	CD4$^+$ cells count at day 30 after HSCT ≥ vs. < median	HR=1.06 (0.24-4.67) p=0.939		
Xu, 2015[97]	PTLD	P & A	CD8$^+$ cells count at day 30 after HSCT ≥ vs. < median	**HR=0.35 (0.17-.72) p=0.004**	**HR=0.34 (0.13-0.92) p=0.033**	
Xu, 2015[97]	PTLD	P & A	CD19$^+$ cells count at day 30 after HSCT	HR=1.26 (0.51-3.10) p=0.621		
Xu, 2015[97]	PTLD	P & A	IgG count at day 30 after HSCT ≥ vs. < median	HR=0.87 (0.30-2.53) p=0.795		
Xu, 2015[97]	PTLD	P & A	IgA count at day 30 after HSCT ≥ vs. < median	HR=0.96 (0.31-3.01) p=0.944		
Xu, 2015[97]	PTLD	P & A	IgM count at day 30 after HSCT ≥ vs. < median	**HR=0.31 (0.11-.88) p=0.027**	**HR=0.27 (0.10-0.75) p=0.012**	
Althubaiti, 2019[31]	PTLD	P	Median of CD20 count	p=0.335	-	
Althubaiti, 2019[31]	PTLD	P	Median of CD19 count	p=0.401	-	
Althubaiti, 2019[31]	PTLD	P	Median of CD4 count	**p=0.003**	-	
Althubaiti, 2019[31]	PTLD	P	Median of CD8 count	**p=0.014**	-	
Althubaiti, 2019[31]	PTLD	P	Median of Gamma delta count	**p=0.004**	-	
Althubaiti, 2019[31]	PTLD	P	Median of NK cells	p=0.250	-	
Althubaiti, 2019[31]	PTLD	P	Median of NKT cells	p=0.112	-	
Althubaiti, 2019[31]	PTLD	P	Median of CD3 count	**p=0.007**	-	
Althubaiti, 2019[31]	PTLD	P	Median of CD8:CD20 ratio	**p=0.007**	-	
Althubaiti, 2019[31]	PTLD	P	CD8:CD20 ratio < 1 vs. CD8:CD20 ratio > 1	**p=0.0003**	-	
			**Transfusion**			
Trottier, 2012[91]	EBV	P	RBC transfusion (Yes vs. No)	NR	HR=2.37 (0.58-9.70)	
Trottier, 2012[91]	EBV	P	RBC transfusion volume (mL) <850 vs. 0	NR	HR=1.99 (0.47-8.44)	**p-value trend= 0.047**
Trottier, 2012[91]	EBV	P	RBC transfusion volume (mL) 850-1890 vs. 0		HR=2.40 (0.56-10.24)	
Trottier, 2012[91]	EBV	P	RBC transfusion volume (mL) >1890 vs. 0		HR=2.86 (0.68-12.11)	
Trottier, 2012[91]	EBV	P	FFP transfusion (Yes vs. No)	NR	HR=1.34 (0.62-2.93)	
Trottier, 2012[91]	EBV	P	FFP transfusion volume (mL) ≤200 vs. 0	NR	HR=0.70 (0.22-2.25)	p-value trend= 0.079
Trottier, 2012[91]	EBV	P	FFP transfusion volume (mL) >200 vs. 0		HR=3.16 (1.00-11.17)	
Trottier, 2012[91]	EBV	P	PLT transfusion volume (mL) 1260-2530 vs. <1260	NR	HR=1.65 (0.86-3.18)	**p-value trend= 0.012**
Trottier, 2012[91]	EBV	P	PLT transfusion volume (mL) >2530 vs. <1260		**HR=2.19 (1.21-3.97)**	
			**Other factors**			
Cesaro, 2010[41]	EBV	P	Period of SCT (1998-2003 vs. 2004-2007)	p=0.8		
Elmahdi, 2016[51]	EBV	P	Year of SCT (After vs. Before 2005)	HR=1.60 (0.53-4.86); p=0.41		
Dumas, 2013[49]	EBV	P & A	History of previous auto-HSCT (Yes vs. No)	**p=0.01**	NS	
Sanz, 2014[87]	EBV	P & A	Prior SCT (Yes vs. No)	NR	NR (NS)	
Garcia-Cadenas, 2015[56]	EBV	A	Prior SCT (Yes vs. No)	**p=0.03**	**HR: 2.6 (1.1-6.4); p=0.04**	
Garcia-Cadenas, 2015[56]	EBV	A	Year of SCT (Before 2010 vs. After 2010)	p=0.1	NS	
Cesaro, 2010[41]	EBV	P	Risk group (Standard risk vs. High risk)	p=0.8		
Elmahdi, 2016[51]	EBV	P	Risk of transplant (High risk vs. Standard risk)	HR=1.29 (0.49-3.40); p=0.603		

Table S4: Risk factors for post-transplant EBV infection and for PTLD explored in the 77 retained studies

First author, year	Outcome	Study population	Risk factors explored	Estimate (95% CI); p-value	
				Univariate results	Multivariate results
Ru, 2020[85]	EBV	P & A	Pretransplant status (Advanced status vs. 1st or 2nd remission)	HR=1.047 (0.881-1.243); p=0.604	NI
Juvonen, 2007[65]	EBV	A	Risk of disease (High risk vs. Low risk)	p=0.35	HR=1.04 (0.60-1.81); P=0.87
Wang, 2019[96]	EBV	P & A	IPSS (Low/Int-2 risk vs. Int-2/High risk)	p=0.147	NI
Wang, 2019[96]	EBV	P & A	AML transformation (Yes vs. No)	p=0.918	NI
Gao, 2019[55]	EBV	P & A	Disease status (CR vs. Not CR)	**p=0.003**	HR=0.6 (0.4-1.1)
Zhou, 2020[100]	EBV	P & A	Disease status before HSCT (Relapse/refractory vs. CR)	HR=2.259 (0.911-5.599); p=0.079	HR=1.279 (0.247-6.629); p=0.769
Liu, 2013[73]	EBV	P & A	Disease status (CR vs. Not CR)	NS	NS
Peric, 2011[84]	EBV	A	Disease status (High risk vs. Standard risk)	p=0.91	-
Peric, 2012[83]	EBV	A	Disease status (Standard risk vs. High risk)	p=0.36	
Garcia-Cadenas, 2015[56]	EBV	A	Comorbitity index (Categories unspecified)	p=0.82	-
Garcia-Cadenas, 2015[56]	EBV	A	EBMT risk score (Categories unspecified)	p=0.56	-
Laberko, 2017[68]	EBV	P & A	Recipient T cell chimerism > vs. < median	p=0.41	-
Sanz, 2014[87]	EBV	P & A	Disease stage (Early, Intermediate, Advanced)	NR	NR (NS)
Patriarca, 2013[4]	EBV	A	Transplant phase (Early vs. Late)	p=0.239	
Patriarca, 2013[4]	EBV	A	Disease status (Resistance and progression vs. Complete and partial response)	p=0.516	-
Wang, 2019[96]	EBV	P & A	Disease progression (Yes vs. No)	p=0.526	NI
Van Esser, 2001[95]	EBV	P & A	Disease status (High risk vs. Standard risk)	HR=1.6 (1.0-2.8); p=0.07	HR=1.4 (0.8-2.6); p=0.2
Dumas, 2013[49]	EBV	P & A	Number of UCB units (Double vs. Single)	p>0.15	-
Peric, 2012[83]	EBV	A	Number of cord blood units (Single vs. Double)	p=1.00	
Cesaro, 2010[41]	EBV	P	Median time to PMN engraftment ≥ vs. <17,5 d	p=0.3	
Cesaro, 2010[41]	EBV	P	Median time to PLT engraftment ≥ vs.<28 d	p=0.9	
Islam, 2010[61]	EBV	P & A	Engraftment: Yes vs. No (Non-malignant group, Malignant group)	(p=1, p=0.49)	-
Burns, 2016[38]	EBV	P & A	Prior rituximab Within 6 months vs. No prior rituximab	HR=0.18 (0.07-0.48); p=0.001	-
Burns, 2016[38]	EBV	P & A	Prior rituximab at any time vs. No prior rituximab	HR=0.34 (0.18-0.64); p=0.001	-
Laberko, 2017[68]	EBV	P & A	Rituximab (Yes vs. No)	p=0.12	HR= 1.12 (0.43-2.86); p= 0.82
Garcia-Cadenas, 2015[56]	EBV	A	Rituximab in the 6 months before transplant (Yes vs. No)	**p=0.02**	NS
Zhou, 2020[100]	EBV	P & A	Early tapering of immunosuppression (Yes vs. No)	HR=1.084 (0.445-2.639); p=0.859	NI
Cohen, 2005[45]	EBV	P	Chimerism (6-week MC vs. 6-week FC)	OR=1.28 (0.43-3.80)	-
Cohen, 2005[45]	EBV	P	Chimerism (12-week MC vs. 12-week FC)	OR=0.94 (0.35-2.5)	-
Van Esser, 2001[95]	PTLD	P & A	A stepwise increase of EBV-DNA by 1 log	NR	**HR=2.9 (1.7-4.8); p<0.001**
Garcia-Cadenas, 2015[56]	PTLD	A	High EBV load (>10000 copies/ml)‡	p=0.8	-
Althubaiti, 2019[31]	PTLD	P	Initial EBV viral load (copies/mL) (Continuous)	p=0.786	-
Althubaiti, 2019[31]	PTLD	P	Maximum EBV viral load (copies/mL) (Continuous)	**p<0.001**	-
Althubaiti, 2019[31]	PTLD	P	EBV viral load >10 000 (copies/mL) (Continuous)	**p=0.039**	-
Pagliuca, 2019[81]	PTLD	P & A	Fever at onset of EBV infection (Yes vs. No)	-	**SHR=6.12 (1.74-21.58); p=0.005**
Fan, 2016[52]	EBV	P & A	ABO blood type mismatched	NR	NR (NS)
Gao, 2019[55]	EBV	P & A	Donor-recipient ABO match (Match vs. Mismatch)	p=0.513	NI
Gao, 2019[55]	PTLD	P & A	Donor-recipient ABO match (Match vs. Mismatch)	p=0.852	NI
Zhou, 2020[100]	EBV	P & A	ABO blood type (incompatibility vs. compatibility)	HR=0.399 (0.142-0.118); p=0.080	HR=0.638 (0.156-2.616); p=0.533
Islam, 2010[61]	EBV	P & A	Survival: Alive vs. Dead (Non-malignant group, Malignant group)	(p=0.66; p=0.41)	-
Fujimoto, 2019[54]	PTLD	P & A	Year of HSCT (2010-2015 vs. 1990-2009)	**HR=2.77 (2.13-3.61); p<0.001**	**HR=1.87 (1.38-2.52); p<0.001**
Garcia-Cadenas, 2015[56]	PTLD	A	Year of SCT (Before 2010 vs. After 2010)	p=0.1	NS
Van der Velden, 2013[94]	PTLD	A	Year of transplant (2006-2008, 2009-2011)	-	NR (NS)
Sanz, 2014[87]	PTLD	P & A	Disease stage (Early, Intermediate, Advanced)	NR	NR (NS)
Uhlin, 2014[93]	PTLD	P & A	Disease stage (Early vs. Late)	NR	NR (NS)
Hoegh-Petersen, 2011[59]	PTLD	A	Disease stage: Poor risk	p=0.11	
Hoegh-Petersen, 2011[59]	PTLD	A	Disease stage: Good risk		

Table S4: Risk factors for post-transplant EBV infection and for PTLD explored in the 77 retained studies

First author, year	Outcome	Study population	Risk factors explored		Estimate (95% CI); p-value	
					Univariate results	Multivariate results
Garcia-Cadenas, 2015[56]	PTLD	A	Comorbidity index (Unspecified)		p=0.4	-
Garcia-Cadenas, 2015[56]	PTLD	A	EBMT risk score (Unspecified)		p=0.69	-
Brunstein, 2006[37]	EBV/PTLD	P & A	Number of donors (2 vs. 1)		-	HR=0.4 (0.1-2.4); p=0.29
Sanz, 2014[87]	PTLD	P & A	Prior SCT (Yes vs. No)		NR	NR (NS)
Garcia-Cadenas, 2015[56]	PTLD	A	Prior SCT (Yes vs. No)		p=0.03	**HR: 6.4 (1.3-31.9); p=0.02**
Landgren, 2009[69]	PTLD	P & A	Second transplantation (Yes vs. No)ǂ		-	**RR=3.5 (1.7-6.3)**
Fujimoto, 2019[54]	PTLD	P & A	Number of allogeneic HSCT (Two or more vs. One)		**HR=2.15 (1.56-2.97); p<0.001**	**HR=1.50 (1.05-2.15); p=0.03**
Garcia-Cadenas, 2015[56]	PTLD	A	Absence of Rituximab prior SCT (Yes vs. No)		p=0.16	-
Cohen, 2005[45]	PTLD	P	Mixed chimaeras (6-week MC vs. 6-week FC)		OR=0.59 (0.07-5.32)	
Cohen, 2005[45]	PTLD	P	Mixed chimaeras (12-week MC vs. 12-week FC)		OR=0.55 (0.11-2.82)	
Althubaiti, 2019[31]	PTLD	P	Median time to 1st EBV		p=0.089	-
Althubaiti, 2019[31]	PTLD	P	Median time from EBV to T-cell subset analysis		p=0.721	-
Kalra, 2018[66]	PTLD	P & A	Time periods (prompt therapy period vs. No EBV monitoring period)		SHR=1.82, p=0.04	SHR=1.34, p=0.06
Gao, 2019[55]	PTLD	P & A	Disease status (CR vs. Not CR)		p=0.413	NI
Liu, 2013[26]	PTLD	P & A	Disease status (CR vs. Not CR)		0.207	0.212
Xuan, 2013[16]	PTLD	P & A	Disease status (CR vs. Not CR)		NS	NS
Xu, 2015[97]	PTLD	P & A	Disease status (High-risk vs. Standard-risk)		HR=0.57 (0.15-2.12); p=0.399	
Uhlin, 2014[93]	PTLD	P & A	Splenectomy (Yes vs. No)		NR	**SHR=4.81 (1.51-15.4); p=0.008**
Uhlin, 2014[93]	PTLD	P & A	MSC treatment		NR	**SHR=3.05 (1.25-7.48); p=0.015**
Wang, 2019[96]	EBV	P & A	RAEB-1 vs. RAEB-2 vs. Other		p=0.244	NI
Wang, 2019[96]	EBV	P & A	Blast (<5% vs. ≥5%)		p=0.222	NI
Zhou, 2020[100]	EBV	P & A	Cystitis (Yes vs. No)		HR=1.987 (0.804-4.912); p=0.137	NI
Liu, 2020[72]	EBV	A	Longer duration of MMF use (until 45-60 days post-transplant) vs. Shorter duration of MMF use (withdrawn by engraftment)		**p=0.033**	-
Liu, 2020[72]	PTLD	A	Longer duration of MMF use (until 45-60 days post-transplant) vs. Shorter duration of MMF use (withdrawn by engraftment)		**p=0.029**	-
Zhou, 2020[100]	EBV	P & A	Third-party cells (Yes vs. No)		HR=0.846 (0.282-2.541); p=0.766	-
Zhou, 2020[100]	EBV	P & A	Well-control of fungus pneumonia pre-SCT (Yes vs. No)		HR=0.339 (0.114-1.008); p=0.052	HR=0.395 (0.068-2.299); p=0.301
Wang, 2019[96]	EBV	P & A	Therapies	DAC+CT vs. Supportive care	p=0.057	HR=2.28; p=0.160
				DAC vs. Supportive care		HR=1.31; p=0.760
				CT vs. Supportive care		HR=2.24; p=0.160

Abbreviations:

A: adults; **AA**: aplastic anemia; **Ag**: antigen; **aGvHD**: acute graft-versus-host disease; **AL**:acute leukemia; **ALG**: antilymphocyte globulin; **ALL**: acute lymphocytic leukemia; **AML**: acute myeloid leukemia; **ANC**: absolute neutrophil count; **ATG**: anti-thymocyte globulin; **ATG-F**: ATG-fresenius; **ATG-T**: ATG-thymoglobulin; **AUC**: area under curve; **auto-HSCT**: autologous hematopoietic stem cell transplantation; **BEAM**: carmustine with etoposide, cytarabine and melphalan; **BM**: bone marrow; **Bu**: busulfan; **CB**: cord blood; **CI**: confidence interval; **CIC**: conventional-intensity conditioning; **CFU-GM**: granulocyte-macrophage colony-forming unit; **cGvHD**: chronic graft-versus-host disease; **CIC**: conventional-intensity conditioning; **CLL:** chronic lymphocytic leukemia; **CML**: chronic myeloid leukemia; **CMV**: cytomegalovirus; **CR**: complete remission; **CsA**: cyclosporine A; **CT**: chemotherapy; **Cy**: cyclophosphamide; **D**: donor; **DAC**: decitabine; **D/R**: donor/recipient; **EBMT**: European Group for Blood and Marrow Transplantation; **EBV**: Epstein-Barr Virus; **FC**: full chimeras; **FFP**: fresh-frozen plasma; **Flu**: fludarabine; **GvHD**: graft-versus-host disease; **HIDT**: haplo-identical donor transplantation; **HL**: Hodgkin lymphoma; **HLA**: human leukocyte antigen; **HLAIDSIB**: HLA identical sibling; **HR**: hazard ratio; **HSCT**: hematopoietic stem cell transplantation; **IPSS**: International Prognostic Scoring System; **KIR**: killer cell immunoglobulin-like receptor; **LFI:** limited field irradiation; **MAC**: myeloablative conditioning; **MC**: mixed chimeras; **MDS**: myelodysplastic syndrome; **Mel**: melphalan; **MFD**: matched family donor; **MM**: multiple myeloma; **MMF**: mycophenolate mofetil; **MMFD:** mismatched family donor; **MMUD:** mismatched unrelated donor; **MMRD**: mismatched related donor; **MNC**: mononuclear cells; **MoAb**: monoclonal antibody; **MPA:** mycophenolic acid; **MPD:** myeloproliferative disease; **MRD**: matched related donor; **MSC:** mesenchymal stromal cells; **MSDT**: matched sibling donor transplantation; **MTX**: methotrexate; **MUD**: matched unrelated donor; **MUDT**: matched unrelated donor transplantation; **NHL**: Non-Hodgkin lymphoproliferative disease; **NI**: Not included; **NK:** natural killer cells; **NKT**: cells, natural killer T cells; **NMAC:** Nonmyeloablative conditioning; **NR**: not reported; **NS**: not significant; **NST:** needing systemic therapy; **OR**: odds ratio; **P**: pediatric; **P & A**: pediatric and adult; **PB**: peripheral blood; **PBSC**: peripheral blood stem cells; **PID**: primary immunodeficiency; **PLT**: platelets; **PMN**: polymorphonuclears; **PTLD**: post-transplant lymphoproliferative disorders; **R**: recipient; **RAEB**: refractory anemia with excess blasts; **r-ATG**: rabbit ATG; **RBC**: red blood cell; **RIC**: reduced-intensity conditioning; **RR:** relative risk; **SAA**: severe aplastic anemia; **SCT**: stem cell transplant; **SHR**: subhazard ratio; **SIB**: sibling; **SRBC:** sheep red blood cell; **TBI**: total body irradiation; **TCD**: T-cell depletion; **TLC**: total lymphocyte count; **TNC**: total nucleated cells; **UCB**: umbilical cord blood; **VP16:** etoposide; **vs.**: versus; +: positive; -: negative.

ǂTime-dependent covariate.

‡normal group was defined by T-cell subsets, B-cells, or serum immunoglobulins within their reference ranges, and the abnormal group was defined by levels outside the reference ranges.
